# Generation of Neural Organoids and Their Application in Disease Modeling and Regenerative Medicine

**DOI:** 10.1002/advs.202501198

**Published:** 2025-05-24

**Authors:** Ruiqi Huang, Feng Gao, Liqun Yu, Haokun Chen, Rongrong Zhu

**Affiliations:** ^1^ Key Laboratory of Spine and Spinal Cord Injury Repair and Regeneration of Ministry of Education, Department of Orthopedics, Tongji Hospital affiliated to Tongji University, School of Life Science and Technology Tongji University Shanghai 200065 China; ^2^ Frontier Science Center for Stem Cell Research Tongji University Shanghai 200065 China; ^3^ Clinical Center for Brain and Spinal Cord Research Tongji University Shanghai 200065 China

**Keywords:** disease modeling, human nervous system, neurodevelopment, neural organoid, stem cells

## Abstract

The complexity and precision of the human nervous system have posed significant challenges for researchers seeking suitable models to elucidate refractory neural disorders. Traditional approaches, including monolayer cell cultures and animal models, often fail to replicate the intricacies of human neural tissue. The advent of organoid technology derived from stem cells has addressed many of these limitations, providing highly representative platforms for studying the structure and function of the human embryonic brain and spinal cord. Researchers have induced neural organoids with regional characteristics by mimicking morphogen gradients in neural development. Recent advancements have demonstrated the utility of neural organoids in disease modeling, offering insights into the pathophysiology of various neural disorders, as well as in the field of neural regeneration. Developmental defects in neural organoids due to the lack of microglia or vascular systems are addressed. In addition to induction methods, microfluidics is used to simulate the dynamic physiological environment; bio‐manufacturing technologies are employed to regulate physical signaling and shape the structure of complex organs. These technologies further expand the construction strategies and application scope of neural organoids. With the emergence of new material paradigms and advances in AI, new possibilities in the realm of neural organoids are witnessed.

## Graphical abstract

Neural organoids provide a versatile platform for neurological research. Advances in organoid technology have partially achieved human neural tissue complexity in terms of tissue structure, cell diversity, and neural signaling, offering insights into neural disorders and regenerative strategies. Technology advances from biomaterials, bio‐manufacturing, and AI expand their applications, paving the way for groundbreaking discoveries in neuroscience.

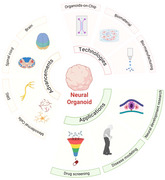

## 1 Introduction

The human central nervous system (CNS) is one of the most complex organs, comprising distinct functional regions of the gyrencephalic brain and 31 segments of the spinal cord. The formation of such intricate tissue structures is induced by the spatiotemporal gradients of morphogens during fetal development. Deficiencies in the proliferation, differentiation, migration, and synaptogenesis of neural progenitor cells (NPCs) during this critical period can lead to neurodevelopmental disorders.^[^ ^1^, ^2^, ^3^ ^]^ Furthermore, this delicate system is highly susceptible to factors that can disrupt neural function. Researchers' understanding of the human CNS mostly comes from cadaver pathological anatomy, which cannot reflect neural activity and signal transduction in a physiological state.^[^ ^4^, ^5^ ^]^ The limitations of traditional 2D cell cultures and animal models further highlight the necessity for advanced in vitro models. Although monolayer cultures are suitable for studying cell function and communication mechanisms, they fail to recapitulate the complex cellular interactions, tissue architecture, and physiological functions of the human CNS. Animal models often exhibit species‐specific differences that hinder their translational relevance to human diseases. Rodents, as the widely employed model organisms in neurodevelopment and disease research, their simplicity in brain size, spinal segment count, structural organization, cellular composition, and intercellular interactions that limit their capacity to accurately reflect the characteristics of the human CNS.^[^ ^6^, ^7^, ^8^, ^9^ ^]^ These limitations have driven the development of more sophisticated in vitro systems capable of mimicking human biology more accurately.

Organoid technology derived from human embryonic stem cells (hESCs) or induced pluripotent stem cells (iPSCs) offers a robust 3D model in a plate for studying the human CNS. In addition to iPSCs and hESCs, other stem cell types, such as reprogrammed adult cells^[^ ^10^ ^]^ and primary cells,^[^ ^11^ ^]^ have also been utilized to generate organoids, further expanding the versatility of these models. These organoids exhibit self‐organization and can represent the cellular diversity and structural complexity of the early human neural tissue compared to monolayer cultured neural cells. Neural organoids that can represent multiple brain regions, including the neocortex, midbrain, and hippocampus, as well as specific spinal cord segments such as the cervical and thoracic regions, have been successfully generated.^[^ ^12^, ^13^ ^]^ Furthermore, innovative techniques have been employed to optimize these models.^[^ ^14^ ^]^ Neural organoids can simulate human brain development, including NPC differentiation, neuronal formation, and neural network establishment. They not only reproduce the brain's morphological features but also exhibit functional neural activities, making them an ideal platform for studying neural network formation and functionality. Neural organoids hold great potential for disease modeling. Organoids generated from patient‐derived stem cells can model the pathological processes of various neurological disorders, such as autism, Alzheimer's disease, and Parkinson's disease, and help elucidate the molecular mechanisms underlying these diseases.^[^ ^15^ ^]^ Neural organoids provide a highly physiologically relevant in vitro platform for drug screening and toxicity testing, thereby enhancing the efficiency and precision of drug screening.^[^ ^16^ ^]^ Using chimeric organoids, researchers can dissect how genetic variations shape the molecular trajectories of neurodevelopment and investigate the interplay between genetics and the environment.^[^ ^17^ ^]^ This research provides critical support for the advancement of personalized medicine. Additionally, Neural organoids can also be used to investigate the unique features of human brain development and differences from other species. For instance, by comparing human and nonhuman primate organoids, researchers can elucidate the evolutionary mechanisms of brain development.^[^ ^18^ ^]^ Neural organoids offer novel tools and perspectives for basic research, disease modeling, drug development, and personalized therapy in the field of neuroscience.

In this review, we summarize the generation methods of neural organoids inspired by human neurodevelopmental patterns, including the design of region‐specific organoids and those integrated with vascular structures or possessing immune functionalities. We also discuss the latest advancements in utilizing neural organoids for disease modeling and the application of transplantation. As the protocols for generating neural organoids continue to improve, we highlight how technologies such as organ‐on‐a‐chip and tissue engineering can address the limitations of traditional approaches, enhancing their potential as refined in vitro models. Finally, we explore how emerging paradigms in functional materials and advancements in artificial intelligence (AI) may offer new directions for the field of neural organoids.

## 2 Development of the Neural Tube and Central Nervous System Formation

The human nervous system is a highly complex and organized structure, shaped by spatiotemporal gradients of various morphogens that guide cell fate determination and neural tube morphogenesis during its development. Neural organoid protocols have been inspired by in vivo development of the CNS.

### 2.1 Cellular Dynamics and Structural Development in Neurogenesis

**Figure 1:**
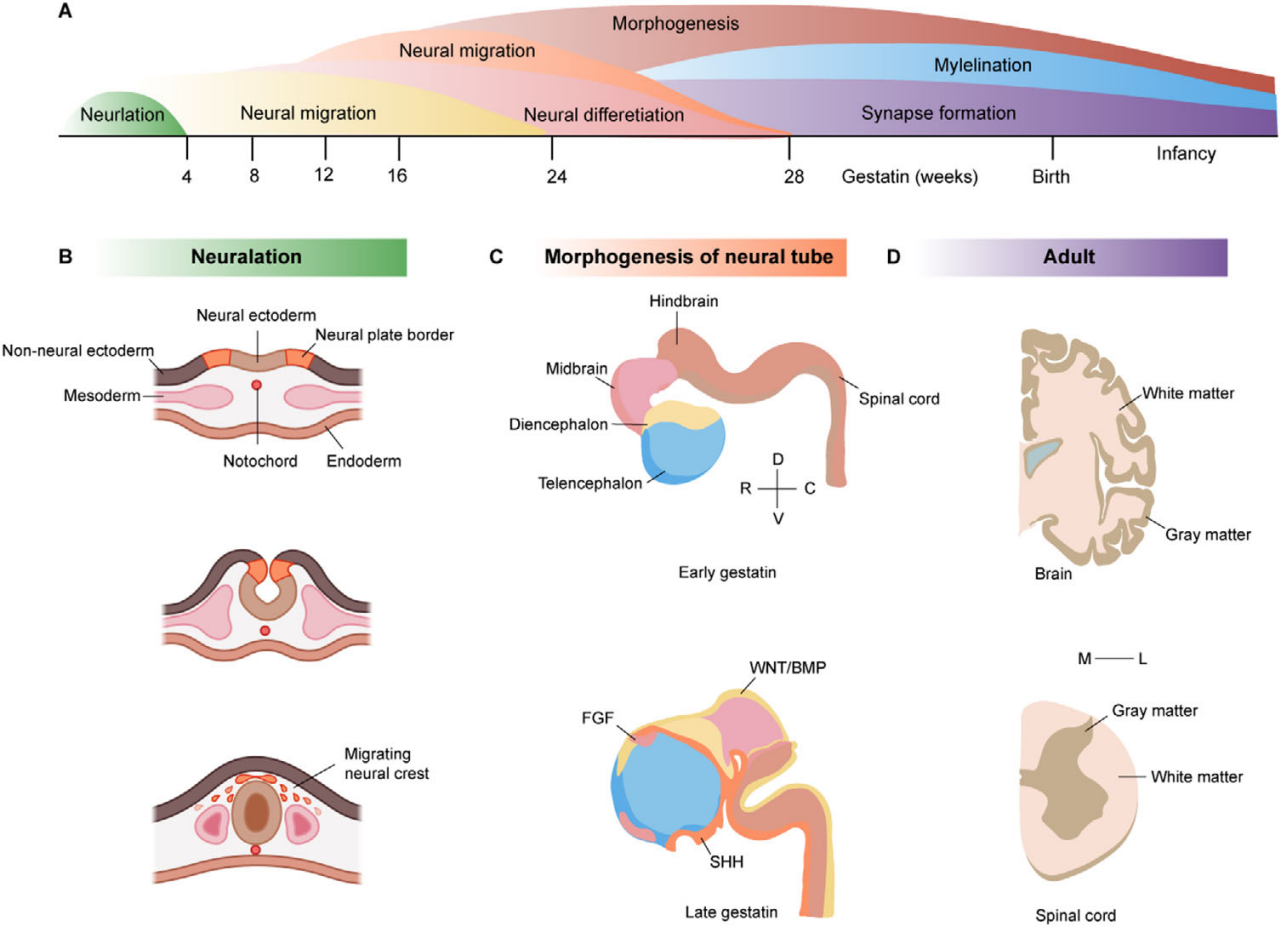
Development of the human CNS. A) Timeline of human neural development. B) Formation of the neural tube. C) Morphogenesis of the neural tube. D) Structure of adult brain and spinal cord. Created by Biorender and Adobe Illustrator 2024.

The formation of the human neural tube is a complex process that involves multiple cellular behaviors, including neural induction, cell proliferation, differentiation, migration, axon growth, and synaptogenesis (**Figure** **1**A). Disrupted cellular behavior can contribute to various neurodevelopmental disorders (**Table** **1**). NT development begins during the trilaminar stage of embryogenesis and arises from the ectoderm (Figure 1B). The notochord, located along the body axis, induces NT formation through the secretion of signaling molecules. The adjacent ectoderm develops into the neural plate. Subsequently, the neural plate undergoes invagination, deepening as embryonic development progresses. The lateral edges of the invaginated neural plate eventually converge at the midline to form the neural tube, which gradually separates from the overlying ectoderm that will give rise to the epidermis. During the separation of the neural tube from the ectoderm, a population of cells forms loose aggregates between the epidermis and the neural tube, known as neural crest (NC) cells. These cells can ultimately form dorsal root ganglia (DRG) and other neural derivatives.^[^ ^19^ ^]^ Following the formation of the neural tube, the neuropores gradually close during embryonic development at Carnegie stage (CS12).^[^ ^20^ ^]^ Failure of this closure can lead to severe neurodevelopmental disorders, such as anencephaly or spina bifida.

**Table 1:** Pathogenic mechanism of neurodevelopmental disorders.

Neurodevelopmental disorders	Pathological feature	Dysfunction of NPCs	Refs.
Microcephaly	Dramatically reduced cerebral cortex size	Reduced proliferation of NPCs during brain development	[21, 22]
Macrocephaly	An increase in brain size or the number of cells	Delayed neuronal differentiation and higher progenitor proliferation	[23]
MDS	Lissencephaly or pachygyria	Reduced proliferation, migration defects, and increased NPC apoptosis	[24]
FXS	Mental retardation syndrome caused by mutations in the FMRP gene present on the X chromosome	Defects of NPCs proliferation and glial cell differentiation	[25]
Down's syndrome	Mental retardation caused by an abnormality in chromosome 21	Decreased proliferative capacity of NPCs, a significant decrease in deep and superficial cortical neuronal cells	[26]
ASD	Language impairment, difficulties in social interaction, and repetitive stereotyped behaviors	Dysregulated NPC proliferation and imbalanced excitatory/inhibitory neurons	[27]

**ASD**, Autism spectrum disorder; **FXS**, Fragile X Syndrome; **MDS**, Miller Dieker syndrome; **NPC**, neural progenitor cell.

The neural tube establishes the major regions of the nervous system along the rostral–caudal (R–C) axis, including the forebrain, midbrain, hindbrain, and spinal cord.^[^ ^28^ ^]^ The formation of the brain is closely linked to the development of the neural tube's anterior region. This process begins with the appearance of narrow rings at the anterior end of the neural tube. Initially, two such rings divide the anterior neural tube into three enlargements known as brain vesicles: the forebrain, midbrain, and hindbrain (Figure 1C). As embryonic development progresses, a new narrow ring forms within the forebrain, further subdividing it into the telencephalon and diencephalon. Similarly, another narrow ring appears in the hindbrain, dividing it into the metencephalon and myelencephalon. Ultimately, the human brain structure develops from these five regions: the telencephalon gives rise to the cerebral cortex, hippocampus, and olfactory bulb; the diencephalon develops into structures such as the retina, thalamus, and hypothalamus; the midbrain vesicle forms the midbrain, including the cerebral peduncles and tectum; the hindbrain differentiates into the cerebellum and pons; and the myelencephalon develops into the medulla oblongata. The caudal end of the neural tube forms the spinal cord.

**Figure 2:**
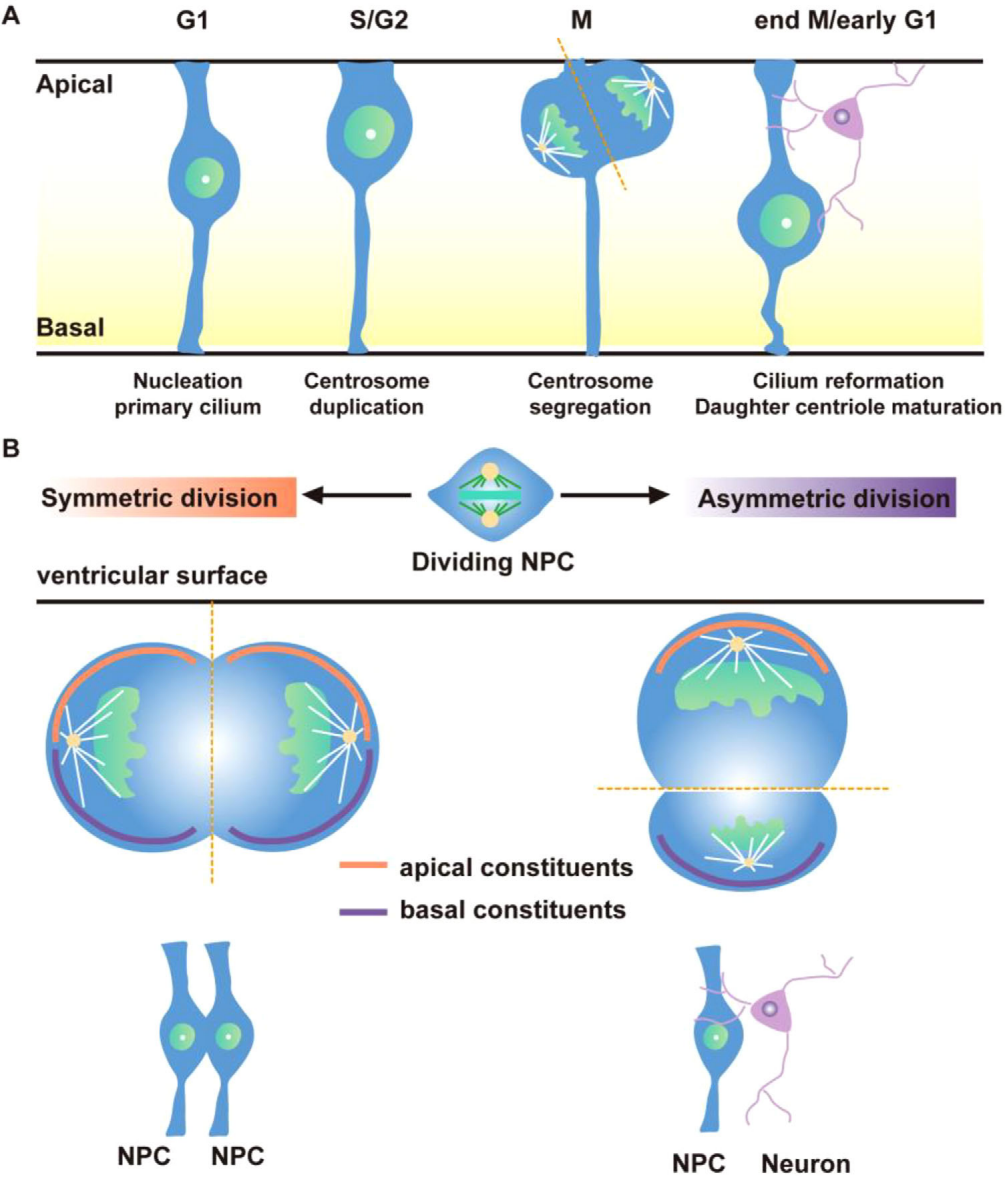
Dividing mode of NPC during development. A) Centrosome cycle in dividing NPCs. B) Symmetrical and asymmetrical divisions of NPCs. Created by Adobe Illustrator 2024.

The development of the CNS involves the proliferation and differentiation of progenitor cells, with a critical balance between these cellular behaviors. Neurons, glial cells, and ependymal cells of the CNS are derived from the neuroepithelium within the neural tube. The neuroepithelium is capable of mitosis. During the interphase, neuroepithelial cells adopt a spindle shape, with their ends extending toward the basal lamina and the lumen. The cell body and nucleus are located near the basal lamina and begin to move toward the lumen, a process known as interkinetic nuclear migration.^[^ ^29^, ^30^ ^]^ This nuclear migration refers to the oscillatory movement of the nucleus between the inner and outer sides of the ventricular zone. During the mitotic phase, the migrating cell body approaches the lumen, and the cell adopts a spherical shape (**Figure** **2**A). Proliferation of NPCs can be categorized into symmetric and asymmetric divisions. Symmetric division produces two identical progenitor cells during the mitotic process; both progenitor cells can independently undergo mitosis, with equal division accompanied by reciprocal movement between the two sides of the ventricular zone. In contrast, asymmetric division typically occurs as progenitor cells begin to differentiate into neurons. In this process, a progenitor cell generates one progenitor cell and one postmitotic neuron, which will no longer divide. This differentiation process is jointly regulated by morphogen and the orientation of the progenitor cells. When the cleavage plane is perpendicular to the ventricular surface, the progenitor cell divides into two identical cells; when the cleavage plane is parallel to the ventricular surface, it divides into one NPC and one postmitotic neuron(Figure 2B).^[^ ^31^ ^]^

### 2.2 Patterning of the Neural Tube

The patterning of the neural tube refers to the process by which it undergoes differentiation and development in a specific manner, guided by distinct genetic regulatory programs. After mitosis, NPCs differentiate into various types of neurons, ultimately forming the complex 3D structure of the CNS. During this process, the spatiotemporal gradients of morphogens precisely regulate cell fate.

The patterning of the neural tube primarily involves the establishment of the R–C axis and the dorsoventral (D–V) axis (Figure 1C). The D–V axis is formed by the interplay of dorsal Bone morphogenetic proteins 4 (BMP4) and Wnt signaling molecules, along with ventrally secreted Sonic hedgehog (SHH).^[^ ^13^ ^]^ The cross‐gradient of these signaling molecules leads to the differentiation of the ventral subpallium (high expression of DLX2/NKX2‐1) from the dorsal pallium (high expression of PAX6).^[^ ^32^ ^]^ In the spinal cord, high expression of PAX6 and PAX3 in the dorsal region and high expression of NKX6‐1, Olig2, and Foxa2 in the ventral region can be detected.^[^ ^33^ ^]^ These transcription factors guide the differentiation of progenitor cells into region‐specific neurons. These morphogens also regulate the specialization of the R–C axis and other cellular behaviors. SHH signaling not only promotes the fate of ventral neurons but also regulates the proliferation of NPCs and guides axon growth.^[^ ^34^ ^]^ Wnt signaling plays a role in neural induction and posteriorization, facilitating the formation of NC cells, regulating the proliferation and differentiation of neural progenitors, and participating in axon guidance and synaptogenesis.^[^ ^28^ ^]^ BMPs promote dorsal neuronal fate while inhibiting neural induction, influencing the formation of NC cells, as well as the differentiation of glial cells and neuronal subtypes.^[^ ^35^ ^]^ At the anterior end of the neural tube, fibroblast growth factor (FGF) and retinoic acid (RA) signaling molecules collaboratively promote anterior neural fates, stimulating neurogenesis and gliogenesis while participating in axon guidance and synaptogenesis.^[^ ^36^, ^37^ ^]^ In the posterior neural tube, which develops into the spinal cord, the cross‐gradient of RA at the anterior end and FGF8/GDF11 at the posterior end induces the formation of the R–C axis. During neural tube development, the forebrain and midbrain specifically express the transcription factor OTX2, while FGF8 is expressed at the boundary between the midbrain and hindbrain.^[^ ^32^ ^]^ The hindbrain and spinal cord exhibit overlapping expression of Homeobox (Hox) genes along the anteroposterior (A–P) axis, with Hox 1–5 expressed in the hindbrain, Hox 4–7 in the cervical spinal cord, Hox 8–9 in the thoracic spinal cord, and Hox10‐11 in the lumbar spinal cord.^[^ ^38^ ^]^

The patterning of the neural tube also involves the migration of neural cells, which is fundamental for the layering of the cerebral cortex and the development of the spinal cord. Neurogenic and gliogenic cells migrate outward from the neuroepithelium to the subpial region, forming a dense cell area known as the marginal zone, while the peripheral sparse cell region develops into the cortical layer. In the spinal cord, the marginal zone develops into gray matter, whereas the peripheral sparse cell region becomes white matter (Figure 1D).

## 3 Advancements in the Construction of Diverse Neural Organoids

### 3.1 Construction of Ectoderm‐Derived Neural Organoids

Restricted by the limitations of the CNS complex organization and ethics, neuroscience research related to the development and diseases of the CNS can only be conducted by using model organisms.^[^ ^39^ ^]^ However, significant species differences between humans and these model animals hinder the ideal simulation of developmental processes and disease mechanisms of the nervous system. The ectoderm is the foundation for the development of both the central and peripheral nervous systems. With advancements in stem cell technology, researchers can now induce the directional differentiation of hESCs and iPSCs by applying specific clues to precisely regulate developmental signaling pathways.^[^ ^40^, ^41^, ^42^ ^]^ This process effectively simulates the development and maturation of the early embryonic nervous system, thereby establishing an in vitro model of the human nervous system.^[^ ^43^ ^]^

Initially, researchers utilized 2D models to culture both mature and progenitor neurons differentiated from human pluripotent stem cells (hPSCs).^[^ ^44^ ^]^ While the 2D culture model provides a solid foundation for studying embryonic development processes and neurodevelopmental diseases, it falls short in accurately replicating the complex morphology and functionality of real tissues, as actual organs comprise a diverse array of cell types. Consequently, researchers developed organoid technology, which enables the arrangement of tissue structures by integrating 2D neuroectodermal cell induction techniques with 3D embryoid body (EB) technology.^[^ ^45^, ^46^ ^]^

**Figure 3:**
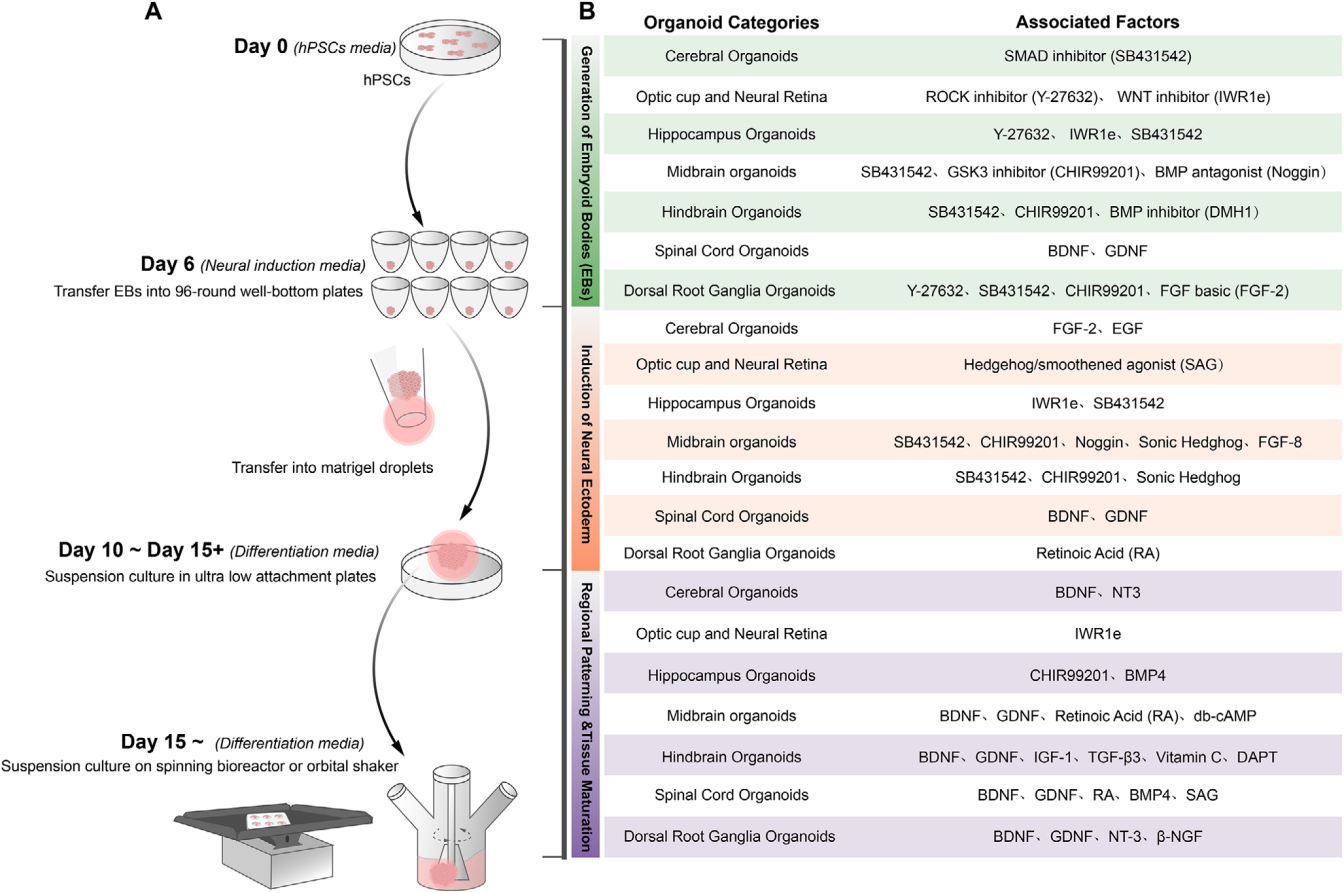
Construction processes of neural organoids derived from pluripotent stem cells. A) The overall strategy to generate neural organoids. B) Overview of the molecules and factors utilized at various stages in the construction process of different types of neural organoids. Created by Adobe Illustrator 2024.

Constructing a 3D culture system from iPSCs typically involves three key steps: neural induction, self‐organization, and regional patterning (**Figure** **3**A). We discuss a range of methods for developing ectoderm‐based nervous system organoids, which include brain organoids derived from various brain regions, spinal cord organoids (SCOs), and peripheral nervous system organoids. Figure 3 provides a basic overview of different type neural organoids construction process.

#### 3.1.1 Generation of Brain Organoid

##### Cerebral Organoids

Leveraging the self‐assembly capabilities and intrinsic signaling of iPSCs, Eiraku et al. utilized serum‐free suspension‐cultured embryonic bodies in conjunction with various neural differentiation‐inducing factors to construct a neuroectoderm‐like structure comprising cortical neuron cell types. This approach facilitated the formation of a 3D cerebral cortex‐like structure through self‐organization.^[^ ^47^, ^48^ ^]^ Under extracellular matrix (ECM)‐free conditions with minimal modeling factors, Sergiu Paşca et al. cultured human cortical spheroid neural structures derived from iPSCs that included both deep and superficial cortical neurons.^[^ ^49^ ^]^ Subsequently, Lancaster et al. developed a 3D organoid culture system derived from hPSCs, characterized by heterogeneous structures across multiple regions and unique human brain features, including the inner fiber layer (IFL) and the outer subventricular zone (OSVZ). This system effectively mimics the developmental characteristics of the human cerebral cortex, such as specific progenitor cell regional organization and a rich population of outer radial glial stem cells (oRGs). By utilizing a rotating bioreactor and incorporating ECM, specifically Matrigel, this system enhances the efficiency of neuronal cell layer polarization and neuroepithelial sprouting, enabling the long‐term survival and culture of organoids for at least ten months.^[^ ^50^ ^]^

##### Forebrain Organoids

The forebrain is composed of two main structures: the telencephalon and the diencephalon. The telencephalon encompasses the olfactory bulb, cerebral cortex, hippocampus, and basal ganglia, while the diencephalon includes the retina, thalamus, and hypothalamus.^[^ ^51^ ^]^ Sasai's team first proposed that pluripotent cells have the potential to differentiate and self‐organize into highly complex and organized structures under 3D‐cultured conditions, thereby mimicking the development of normal tissues and organs.^[^ ^52^ ^]^ This type of organoid construction method, without the involvement of exogenous model factors, is called unguided differentiation, which mainly relies on the inherent self‐organizing program and differentiation capabilities of the cells, allowing for the generation of various cell lineages, including the forebrain, midbrain, hindbrain, and choroid plexus.^[^ ^48^ ^]^ A culture technique called SFEBq (serum‐free floating culture of EB‐like aggregate with quick reaggregation) can generate self‐organized multilayer optic cup tissue in vitro. This research highlights the roles of the FGF and WNT/β‐catenin signaling pathways in the differentiation process of optic tissue.^[^ ^53^ ^]^ In 2011, researchers reduced neural tube tailing by inhibiting the WNT pathway and subsequently activated both the WNT and SHH pathways to promote the formation of retinal epithelial structures. They discovered that hESC‐derived retinal epithelium could self‐assemble into hemispherical epithelial vesicles, forming patterns along the proximal‐distal axis of the vesicles.^[^ ^54^ ^]^

The un‐guided differentiation method mimics the natural developmental processes of cells in vivo while minimizing excessive exogenous interference. However, organoids generated through this method present limitations, such as high variability and heterogeneity, and they fail to replicate the diverse subregions of the human brain accurately. Researchers incorporate exogenous patterning factors to produce more refined brain region organoids, thereby directing their development toward specific cell lineages. In recent years, researchers have successfully generated region‐specific organoids for the retina, hippocampus, thalamus, and hypothalamus (**Table** **2**). Nakano et al. developed a protocol for inducing hESCs into retinal tissue that generates a typical three‐layered retinal structure.^[^ ^55^ ^]^ Based on this protocol, the researchers improved the organoid culture methods through chemical modification, ECM modification, and co‐culture techniques, leading to retinal organoids with improved differentiation efficiency and enhanced photoreceptor maturity.^[^ ^56^, ^57^, ^58^ ^]^ Elke Gabriel et al. overcame the technique limitations in the generation of brain and retinal organoids separately by constructing optic vesicle‐containing brain organoids (OVB organoids) for the first time. Between days 20 and 30, pigmented dots were observed on one side of the organoids, indicating the formation of forebrain organoids with primitive eye fields. Additionally, researchers discovered that long‐term cultured OVB organoids can generate light‐sensitive and complementary optic vesicle (OV) cell types, including primitive corneal epithelial cells, retinal pigment epithelium, and axon projections. This model provides a valuable resource for investigating human brain and eye development, as well as for modeling diseases.^[^ ^59^ ^]^ In 2015, researchers induced BMP and WNT signaling to generate hESCs‐derived choroid plexus, the most dorsomedial part of the forebrain. They achieved self‐organization of medial forebrain tissue by modulating the exposure of patterning factors, which successfully resulted in the generation of choroid plexus, cortical hem, and medial pallidum tissue. After isolation of the dorsomedial forebrain‐like tissues, the generation of Zbtb20^+^/Prox1^+^ granule neurons and Zbtb20^+^/KA1^+^ pyramidal neurons exhibiting electrophysiological functions can be observed after long‐term culture.^[^ ^60^ ^]^ This research recreated the in vivo development process of the human hippocampus, providing a novel experimental platform for investigating hippocampal‐related diseases. Xu et al. reprogrammed astrocytes from the brain tissue of focal cortical malformation (FCD) patients into induced iPSCs, and further induced the generation of dorsal and ventral forebrain organoids (DFOs and VFOs). This study presents a method for the generation of patient‐specific, region‐specific forebrain organoids that effectively simulate the heterogeneous pathological characteristics of FCD, thereby providing a valuable platform for the development of precise therapeutic strategies.^[^ ^61^ ^]^

**Table 2:** Research in forebrain organoid construction.

Organoid type	Summary of key elements	Findings	Refs.
Cortex organoids	An improved SFEB method to differentiate functional cortical neurons from hESCs reveals the ability of hESCs‐derived cortical neuroepithelium in self‐organizing tissue formation.	Controlling the regional identity of hESC‐derived cortical and non‐cortical tissues by manipulating exogenous signals is crucial for understanding brain development. FGF‐8 induces the formation of forebrain regions, while WNT and BMP signaling promote the formation of hindbrain regions.	[47]
Cortex organoids	Established a new 3D brain organoid model–sliced neocortical organoids (SNOs) that can simulate the development process of the human cerebral cortex	Through slicing methods, SNOs overcome diffusion limitations, prevent cell death, and form distinct upper and deep cortical structures during long‐term culture. iPSC‐derived SNOs from psychiatric patients harboring DISC1 gene mutations reveal defects in cortical neuron subtype fate determination.	[68]
Self‐assembled dorsal telencephalon organoids	Generated 3D self‐organizing structures recapitulated the early human dorsal telencephalon developmental program.	Constructed 3D aggregates with forebrain fate by using two hiPSC lines (PGP1‐1 and i03‐01#9) in which TBR2‐expressing interneuron precursor cells were identified, along with GABAergic progenitor cells and GAD‐67^+^ inhibitory neurons observed on day 50 of differentiation.	[69]
Dorsal and ventral forebrain organoids	Generated personalized dorsal and ventral forebrain organoids (DFOs and VFOs) from brain astrocytes from focal cortical dysplasia (FCD) type II patients, which successfully replicated pathological features of FCD.	Patient‐derived DFOs exhibit abnormal cell proliferation and morphological structures, including the presence of malformed neurons and balloon cells, while VFOs exhibit abnormal cell types and neuronal migration. Compared with the normal group, it was found that patient DFOs and VFOs showed significant gene expression differences in cell cycle, neuronal markers, and cardiomyocyte development.	[61]
Optic cup tissue	Transcriptome analysis of self‐organized eye tissues revealed the gene expression pattern during the development of eye tissue which provides molecular clues for studying the development of vision‐related areas in forebrain organoids.	Generated complex, multi‐layered self‐assembled eyecup structures through SFEBq culture technology. And analyzed the role of FGF or WNT/β‐catenin signaling pathway in the differentiation process of eye tissue by RNA‐Seq.	[70]
Optic vesicle‐containing forebrain organoids (OVB organoids)	Achieved joint construction of forebrain organoids and eye primordium.	Constructed the forebrain organoids containing multiple complementary OV cell types to model retinal development and function, and utilized the OVB organoids to investigate the interactions between the retina and the brain.	[71]
Hippocampus organoids	Developed a method to generate functional hippocampal neurons from dorsomedial forebrain tissue derived from human embryonic stem cells.	BMP and WNT signaling are utilized to induce the choroid plexus, while the self‐organization is achieved by modulating the exposure to BMP and WNT signaling. Prolonged isolation and culture of these forebrain tissues resulted in the formation of a network comprising Zbtb20^+^/Prox1^+^ granule neurons and Zbtb20^+^/KA1^+^ pyramidal neurons, both exhibiting distinct electrophysiological functions.	[60]
Choroid plexus (ChP) organoids	Established an in vitro model capable of simulating the cerebrospinal fluid secretion and barrier formation functions of ChP.	By using BMP4 and CHIR to induce hPSCs‐derived ChP organoids with cystic structures containing colorless fluid. Utilizing this model as a tool to predict drug CNS permeability.	[72]
Arcuate nucleus organoids (ARCOs)	Induced the generation of functional hypothalamic arcuate nucleus‐like organs	Generated the ARCOs from human iPSCs through specific induction and differentiation steps. PWS patients’ iPSCs derived‐ARCOs revealed the cellular and molecular defects of PWS in the early developmental stages, providing a new perspective for understanding the pathophysiology of PWS.	[62]
Ventral thalamic organoids (vThOs)	Constructed ventralized human thalamic organoids containing the thalamic reticular nucleus.	Induced the formation of the ventral thalamus by adding recombinant SHH protein at a specific developmental stage (days 14 to 22), and discovered the important roles of PTCHD1 and ERBB4 in the development of TRN neurons by using the vThOs.	[67]
Hypothalamic‐pituitary unit organoids	By optimizing iPSC differentiation conditions, generated functional hypothalamic–pituitary hybrid organoids from human iPSC, successfully recapitulating the hypothalamic–pituitary development process.	By optimizing factors such as cell number, KSR, BMP4, and SAG concentration, and BMP4 addition timing, the proportion of ACTH^+^ cells was increased, and the ACTH secretion capacity of induced pituitary cells was equivalent to that of adult mouse pituitary glands. Simultaneously, developing anterior pituitary and hypothalamic neurons were generated in prolonged culture. Around day 200, CRH^+^ cells appeared in the inner layer of the aggregates (hypothalamic region), indicating maturation of hypothalamic‐like cells.	[73]

SFEB, serum‐free floating culture of EB‐like aggregate with quick reaggregation; hESC, human embryonic stem cell; FGF‐8, fibroblast growth factor; BMP, Bone morphogenetic proteins; SNOs, brain organoid model–sliced neocortical organoids; iPSC, induced pluripotent stem cell; hiPSCs, human induced pluripotent stem cells; 3D, three‐dimensional; DFOs and VFOs, dorsal and ventral forebrain organoids; FCD, focal cortical malformation; RNA‐Seq, RNA sequencing; OV, optic vesicles; OVB, optic vesicles‐containing forebrain organoid; ChP, Choroid plexus; CNS, central nervous system; PWS, Prader‐Willi syndrome; SHH, Sonic hedgehog; PTCHD1, patched domain containing 1; TRN, thalamoreticular nucleus; KSR, knockout serum replacement; SAG, smoothened agonist; ACTH, adrenocorticotropic hormone; CRH, corticotropin‐releasing hormone.

By further adjusting the timing of model formation and the combinations of inductive factors, it is possible to obtain a more refined specific brain regions modeling. Recently, significant progress has been achieved in the development of brain organoids that effectively replicate specific subregions of the human brain. The first arcuate nucleus organoids (ARCOs) were generated from iPSCs by activating the SHH pathway and inhibiting the WNT signaling pathway.^[^ ^62^ ^]^ This study integrated transcriptomic data from ARCOs derived from iPSCs of patients with Prader‐Willi syndrome (PWS), revealing that these organoids exhibit abnormal differentiation patterns and functional defects, including impaired leptin response and exacerbated inflammatory responses. This research highlights the potential of organoid technology to replicate complex brain regions and associated diseases, thereby providing a valuable tool for investigating the development of the human arcuate nucleus and elucidating the mechanisms underlying related disorders. The thalamus is divided into ≈60 nuclei, with the thalamoreticular nucleus (TRN) serving as a key component of the ventral thalamus. The TRN filters and modulates the flow of information from the thalamus to the cerebral cortex, playing a crucial role in processes such as attention, social memory, and sleep rhythms.^[^ ^63^, ^64^ ^]^ Dysfunction of the TRN has been linked to various neurodevelopmental and psychiatric disorders, including autism spectrum disorder, attention‐deficit/hyperactivity disorder, and schizophrenia.^[^ ^65^, ^66^ ^]^ Researchers transformed hESCs into ventral thalamic organoids (vThOs) with distinct nuclear characteristics by introducing recombinant SHH proteins at specific developmental stages. The vThOs exhibited molecular and functional characteristics analogous to those of the ventral thalamus, including high expression levels of GABAergic neuron markers and the generation of high‐frequency action potentials. Additionally, the researchers employed CRISPR interference technology (CRISPRi) to inhibit the expression of TRN‐specific, disease‐related genes, discovering that *PTCHD1* and *ERBB4* play crucial roles in TRN development, with their functional disruption adversely affecting neuronal function. This study not only provides a method to generate thalamic organoids with specific nuclei characteristics, but also provides new experimental tools and theoretical basis for studying TRN‐related neurodevelopmental disorders.^[^ ^67^ ^]^

##### Midbrain Organoids

Dopamine neurons are mainly derived from the ventral midbrain and are closely associated with neurodegenerative diseases and addiction‐related symptoms. Qian et al. employed 3D printing technology to design a miniaturized spinning bioreactor. Based on this design, they developed a culture program for generating organoids in specific brain regions, including the forebrain, midbrain, and hypothalamus, thereby simulating human brain development and providing a valuable platform for regionalized brain organoid construction.^[^ ^74^ ^]^ Jo et al. treated embryoid bodies (EBs) with dual SMAD inhibitors and WNT pathway activators to facilitate differentiation into neuroectoderm. Subsequently, they introduced SHH and FGF8 to induce differentiation toward a midbrain fate, successfully constructing self‐organizing 3D midbrain‐like organoids (hMLOs) composed of functional midbrain dopaminergic (mDA) neurons, where the presence of neuromelanin (NM)‐like granules was observed.^[^ ^75^ ^]^ In 2024, Kim et al. enhanced the experimental protocols established by Kriks et al. and Jo et al.,^[^ ^75^, ^76^ ^]^ generating midbrain organoids expressing midbrain‐specific neuronal markers, including dopamine synthase (tyrosine hydroxylase, TH) and dopamine transporter (DAT). Notably, exposure to the opioid “fentanyl” caused neurodevelopmental damage and altered neuronal synaptic plasticity of the midbrain organoids, thus providing an effective research platform for investigating the mechanisms of addiction and potential treatments.^[^ ^77^ ^]^

##### Hindbrain Organoids

The hindbrain plays a crucial role in sustaining essential life functions, coordinating movement, and transmitting information. Vasiliki Mahairaki's team isolated peripheral blood mononuclear cells (PBMCs) from both healthy individuals and Alzheimer's disease (AD) patients and reprogrammed them into iPSCs. By activating WNT and SHH signaling pathways, the iPSCs were differentiated into hindbrain organoids containing 5‐HT neurons (5‐HT‐organoids), which were utilized to evaluate differences in therapeutic responses to escitalopram oxalate concerning neuropsychiatric symptoms in AD patients.^[^ ^78^ ^]^ In 2024, Pang et al. developed brain organoids that exhibit the characteristics of the medulla spinal trigeminal nucleus (SpV) through a combination of static and rotating 3D culture, alongside SMAD inhibition and the activation of WNT and retinoic acid (RA) signaling. Single‐cell transcriptome analysis revealed the presence of various cell types within the hindbrain spinal trigeminal organoids (hmSpVOs), including NPCs, intermediate progenitor cells, immature neurons, excitatory neurons, and inhibitory neurons. Neurons in long‐term cultured hmSpVOs demonstrated complex axonal and dendritic structures, as well as the presence of GABAergic and glutamatergic neurotransmitters. Furthermore, the researchers fused hmSpVOs with thalamic organoids (hThOs) to create human fused SpV‐thalamic organoids (hSTOs), effectively simulating the neural circuitry between the SpV and the thalamus.^[^ ^79^ ^]^

Brain organoid technology has provided a revolutionary in vitro model system for neuroscience research. Recent studies have expanded from single‐region brain organoids to heterogeneous multi‐regional brain organoids, aiming to simulate the complex structures and functional connections of the human brain. However, the construction of heterogeneous multi‐regional brain organoids faces multiple technical challenges. Achieving precise control over stem cell differentiation into specific regional identities, as well as guiding their ordered assembly in 3D space to form structurally defined and correctly connected complex structures, remains a significant technical challenge. This requires the delicate modulation of multiple signaling pathways and spatiotemporal cues.^[^ ^80^ ^]^ Furthermore, existing brain organoids often exhibit insufficient maturation, typically recapitulating only early embryonic or fetal developmental stages. These stages frequently lack the complex cellular subtypes found in the adult brain, particularly the diverse inhibitory interneurons and mature glial support systems, such as astrocytes, oligodendrocytes, and microglia. This limitation restricts their ability to model adult brain functions and late‐onset diseases.^[^ ^80^, ^81^ ^]^ The absence of an endogenous functional vascular network results in hypoxia and necrosis in the core regions, which restricts model size and long‐term culture viability, thereby hindering the study of processes dependent on blood circulation, such as the blood–brain barrier and neuroinflammation.^[^ ^50^, ^80^ ^]^ Although Cakir et al. initially established a vascular network in cortical organoids by overexpressing hETV2 in hESCs, the formation of a functional vascular system remains a domain challenge.^[^ ^82^ ^]^ In addition to structural considerations, achieving and validating physiologically relevant long‐range axonal projections and functional synaptic connections between different brain region organoids to replicate the complex topology of in vivo neural circuits remains a critical challenge for functional simulation.^[^ ^83^ ^]^ Concurrently, the inherent variability arising from the self‐organization process leads to significant batch‐to‐batch and even intra‐batch variations in size, shape, cellular composition, and regional architecture.^[^ ^84^ ^]^ The absence of standardized protocols and quality control metrics undermines the reproducibility of research findings and the feasibility of large‐scale applications, such as high‐throughput drug screening.

Despite encountering challenges, significant progress has been achieved in the research of heterogeneous multi‐region brain organoids in recent years. In the field of integrating multi‐regional brain tissue organ technology, researchers have developed the ‘assembloid’ technique, which has successfully fused regional organs, providing a novel model for studying neuronal migration and circuit formation.^[^ ^85^, ^86^ ^]^ Furthermore, by utilizing microfluidic chips to facilitate the formation of bidirectional axonal connections between two brain organoids, this model has been employed to analyze neuronal activity within organoid systems and the functional significance of axonal connections between these organoids.^[^ ^87^ ^]^ The advancement of manufacturing and cultivation technologies has significantly propelled progress in the field of organoid construction. Researchers have leveraged microfluidic technology to create spatially well‐defined multi‐regional structures, enabling precise control over morphogen gradients.^[^ ^88^ ^]^ Additionally, 3D bioprinting technology offers a sophisticated platform for organoid culture and development, providing new tools for investigating human brain development and related diseases. Researchers have constructed a tunable GelMA scaffold using 3D bioprinting technology to regulate the development and patterning of cortical organoids and to support the co‐culture of organoids with vascular cells.^[^ ^89^ ^]^ Cai et al. developed a novel vascular network‐inspired diffusible (VID) stent technology through 3D printing, which addresses the limitations of traditional organoids regarding the diffusion of oxygen, nutrients, metabolites, signaling molecules, and drugs by simulating the diffusion characteristics of physiological vascular networks, thereby enhancing the physiological functions of organoids and improving the accuracy of drug responses.^[^ ^90^ ^]^

Looking ahead, research on multi‐regional brain organoids is set to advance toward greater complexity, enhanced functionality, and broader applications. A primary objective is to increase the physiological fidelity of these models by accurately recapitulating finer anatomical structures. This involves incorporating a more comprehensive range of cell types, including diverse interneuron subtypes, mature astrocytes, and oligodendrocytes, as well as integrating non‐neural components like the choroid plexus and meninges.^[^ ^91^ ^]^ Meanwhile, developing more effective strategies for constructing stable, long‐range, and topologically accurate functional neural circuits is crucial, along with real‐time monitoring and modulation techniques such as optogenetics, multi‐electrode arrays (MEAs), and live‐cell imaging. Overcoming the vascularization bottleneck by achieving controllable and efficient in vitro vascularization to support larger, longer‐lived, and more mature organoids remains a critical task.^[^ ^92^ ^]^ Concurrently, advancing the standardization, automation, and scalable production of culture and assessment protocols will be fundamental for enabling large‐scale applications in drug screening and toxicology testing.^[^ ^93^ ^]^ Building upon these advancements, multi‐regional brain models derived from patient‐specific iPSCs hold significant promise for modeling complex neurodevelopmental disorders, such as autism spectrum disorder, neurodegenerative diseases like Alzheimer's disease and Parkinson's disease, and psychiatric conditions, including schizophrenia, thereby driving progress in personalized medicine.^[^ ^94^, ^95^ ^]^ Lastly, as the complexity and functional capacity of these models increase, associated ethical considerations, such as the potential for consciousness or sentience, require ongoing attention, in‐depth discussion, and the establishment of corresponding ethical guidelines and regulatory frameworks.^[^ ^96^ ^]^

#### 3.1.2 Generation of Spinal Cord Organoid

The spinal cord is a complex tubular structure consisting of numerous neuron subtypes and 31 segments along the A–P axis.^[^ ^97^ ^]^ Prompted by studies of brain organoids, researchers have initiated efforts to generate SCOs. Lippmann et al. successfully induced tissue exhibiting spinal cord characteristics by manipulating the signaling clues of the embryonic neural tube.^[^ ^98^ ^]^ Meinhardt et al. induced individual mouse ESCs to form 3D neuroepithelial vesicles exhibiting clear apical/basal polarity. They subsequently directed these vesicles to the cervical level using RA, further investigating the effects of chemically specific microenvironments on neural tube patterning.^[^ ^99^ ^]^ By activating the BMP4 and SHH signaling pathways, the SFEBq method was employed to induce hiPSCs into dorsal and ventral spinal cord‐like tissues (SCLT) encompassing various spinal neuron subtypes, establishing a novel platform for the investigation of complex spinal cord neural circuits.^[^ ^100^ ^]^ Lee et al. presented a construction method for spinal cord‐like organoids (hSCOs) that simulates early spinal cord induction and neural tube morphogenesis. Researchers induced caudal neural stem cells (cNSCs) to form spheres by administering the WNT activator CHIR99021 and the TGF‐β signaling inhibitor SB431542, followed by the addition of bFGF to expand the arrangement of neural epithelium (NE). After the removal of bFGF, RA was introduced to promote cellular specialization and to simulate the morphogenetic processes of the neural tube, which include the formation of the neural plate, neural folding, and the creation of the neural tube.^[^ ^101^ ^]^ The advancement of spinal cord organoid construction techniques has provided a significant experimental platform for studying human spinal cord development and related diseases. Currently, SCOs are utilized to examine developmental spinal cord diseases, degenerative spinal cord diseases, neuropathic pain, and spinal cord injuries (SCI). These applications contribute to the development of new therapeutic methods and drug‐screening models.^[^ ^10^, ^102^, ^103^, ^104^ ^]^ Xu et al. successfully reprogrammed human astrocytes into early neuroectodermal cells, subsequently generating human astrocyte‐derived organoids (hAD‐Organs). They then activated the FGF, SHH, and BMP4 signaling, inducing the hAD‐Organs to develop into SCOs exhibiting both dorsal and ventral spinal neuron characteristics (hADSC‐Organs). This study demonstrated that the transplanted SCOs possess spinal cord neuron properties and can effectively communicate with host neurons, forming synaptic connections and showing potential for neural repair.^[^ ^10^ ^]^ In 2024, Tiago Rito et al. reported significant advancements in the research of spinal cord organoids. By precisely modulating the WNT, BMP, and NODAL signaling pathways, the researchers successfully developed a 3D model simulating human trunk development that exhibited morphogenetic movements. They generated elongated structures, referred to as notoroids, which contain notochord, ventral neural, and mesodermal tissues that closely resemble the morphology and molecular characteristics of the in vivo notochord. Additionally, the study revealed that the neural tissue adjacent to the in vitro generated notochord cells exhibited molecular characteristics akin to floor plate and ventral neural progenitor cells, suggesting that the generated notochord can pattern surrounding tissues in vitro contexts.^[^ ^105^ ^]^ Nonetheless, current SCOs still exhibit certain deficiencies, including significant heterogeneity and low neural maturity. Future research will focus on enhancing the maturity and functionality of SCOs, as well as generating organoids that incorporate a more comprehensive developmental microenvironment.

#### 3.1.3 Generation of Peripheral Nervous System Organoid

Consisting of nerve trunks, nerve plexuses, ganglia, and nerve terminal devices, the peripheral nervous system (PNS) is responsible for transmitting signals between the CNS and other parts of the body, participating in sensory perception, motor control, and autonomic nervous functions. While neural organoids have been extensively developed and studied, the generation of organoids with PNS characteristics remains a relatively new but rapidly evolving field due to its intricate structural.

Ganglia are clusters of nerve cells in the PNS or CNS that are crucial for the formation of neural networks. Sensory ganglia (SG) comprise various subtypes of neurons, which enables them to respond to stimuli across different sensory modalities.^[^ ^106^, ^107^ ^]^ Research indicates that sensory ganglia are implicated in several neurological diseases, including severe pain syndromes.^[^ ^108^, ^109^, ^110^ ^]^ Xiao et al. employed a combination of the transcription factors Ascl1, Brn3b/3a, and Isl1 (collectively referred to as ABI) to reprogram mouse and human fibroblasts into self‐organizing sensory ganglion organoids (iSG), in which various subtypes of sensory neurons, including nociceptive neurons, thermosensitive neurons, mechanosensitive neurons, and proprioceptive neurons, were detected. Additionally, iSG cells transplanted into the DRG of adult rats were found to maintain the characteristics of sensory neuron subtypes and exhibited neural innervation capabilities.^[^ ^111^ ^]^ Zachary et al. provided a method for constructing elongating multi‐lineage organized (EMLO) gastruloids in which peripheral neurons derived from NC cells formed connections with spinal cord regions and exhibited functional characteristics of motor neurons, sensory neurons, and autonomic neurons. This model successfully simulated the co‐development of the CNS and PNS during early human development and provided new perspectives for studying the interactions between multi‐lineage tissues.^[^ ^112^ ^]^ DRGs originate from NC cells and sequentially differentiate into glial cells and various types of neurons under the guidance of multiple signaling pathways. By analyzing the early differentiation trajectories of human NC cell lineages, researchers discovered that NC cells undergo two critical windows of neurogenesis during their differentiation into DRGs, producing two types of unspecialized sensory neurons (uSN), namely uSN1 and uSN2, which have not yet undergone specific differentiation. Through further research, the researchers identified the external signaling pathways and internal transcription factor regulatory mechanisms involved in the fate of uSNs, successfully constructing DRG organoids (hDRGOs). hDRGOs can simulate the developmental process of human DRGs and produce a DCC^+^/NTRK3^+^/NTRK1^+^ nociceptor subtype that is specifically enriched in human DRGs.^[^ ^113^ ^]^ The transmission and perception of auditory information depend on the physiological interactions between the peripheral and central auditory systems. Researchers induced NPCs isolated from human embryonic cochlea and auditory cortex to generate organoids. Based on super‐aligned carbon nanotube (SA‐CNT) scaffolds, they constructed a human auditory neural circuit model with functional synaptic connections for the first time. This model provides new tools for studying auditory pathway disorders such as sensorineural hearing loss.^[^ ^114^ ^]^

### 3.2 Generation of Neural Organoids Containing Mesodermal Cells

The CNS is composed of neurons and non‐neuronal cells, the non‐neuronal cells including astrocytes, microglia, endothelial cells, and pericytes. These cells undergo coordinated differentiation during embryonic development, providing critical support and nourishment to neurons in adult neural tissue. Although neuroectodermal lineages primarily give rise to neural organoids, recent studies have begun to address the deficit of non‐neuronal ectodermal components in the CNS, particularly focusing on the inclusion of microglia and vascular endothelial cells.

#### 3.2.1 Neural Organoids Containing Microglia

**Figure 4:**
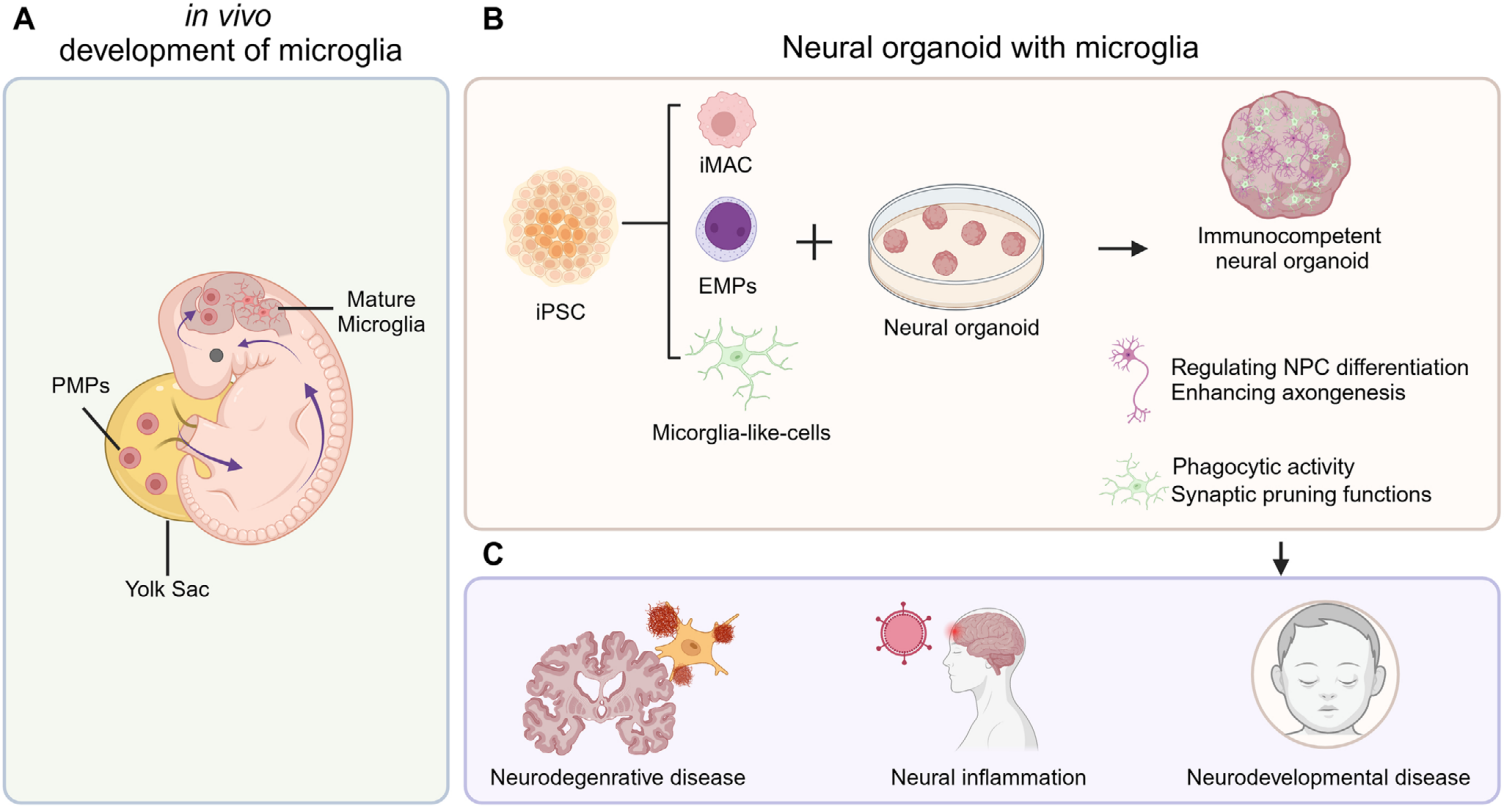
Generation and application of neural organoids containing microglia. A) Development of microglia in human embryo. B) Generation method of neural organoids containing microglia by fusion of microglia progenitors with organoids. C) Application of neural organoids containing microglia in human neurological disease modeling. Created by Biorender.com.

Microglia are the resident macrophages of the CNS, distinct from neurons and astrocytes, as they arise from mesodermal lineages. During early embryonic development of human, primitive macrophage progenitors (PMPs), originating from the yolk sac, migrate to the CNS around gestational week 4.5 via vasculature (**Figure** **4**A). Upon arrival, they interact with NPCs, collaboratively contributing to the development of the nervous system.^[^ ^115^, ^116^ ^]^ In the adult CNS, microglia play a crucial role in regulating synaptogenesis and neurotransmitter release. They are actively involved in the progression of neurodegenerative diseases and neural injury, contributing to disease pathology and circuit remodeling. Therefore, the generation of neural organoids for human disease modeling must take into account of microglia.

Early studies have demonstrated that by modifying the construction protocol of brain organoids, microglia can spontaneously generate within the organoids. Quadrato et al. found that due to the absence of dual‐SMAD inhibition, a limited number of mesodermal lineage cells emerged in cerebral organoids.^[^ ^117^ ^]^ Ormel et al. reported that these mesodermal progenitor cells can differentiate into microglia under the guidance of the microenvironment provided by neuroectodermal cells in cerebral organoids (COs).^[^ ^118^ ^]^ Meanwhile, another research group observed that in retinal organoids generated by an unguided differentiation protocol, microglia aggregate in non‐pigmented, 3D cystic compartments. Additionally, low doses of BMP4 signaling were found to inhibit the differentiation of goblet cells while promoting the differentiation of microglia and the formation of cysts.^[^ ^119^ ^]^ Despite the presence of characteristic morphology and the expression of specific markers of microglia in neural organoids, as well as their roles in mediating inflammatory responses and phagocytosis, there are limitations regarding the variability in both the quantity and distribution of microglia among individual organoids. Furthermore, the absence of SMAD inhibition introduces issues of heterogeneity in neural development.^[^ ^118^ ^]^

To obtain reproducible and more homogeneous microglia‐containing neural organoids, researchers have made significant efforts (Figure 4B). Multiple research teams have induced iPSCs to generate microglia‐like cells, which were then directly fused with brain organoids. This approach resulted in phenotypes similar to those of in vivo microglia, which respond to and aggregate in areas of physical neural injury within the organoids, exhibiting amoeboid morphology.^[^ ^120^, ^121^ ^]^ Xenotransplantation of microglia into cerebral organoids can induce transcriptional changes in neural stem cells and modulate synaptic density, thereby regulating neural signaling within the organoids.^[^ ^122^ ^]^ While mature microglia or microglia‐like cells can effectively integrate into neural organoids and perform functional roles, they do not replicate the synchronized development of PMPs and NPCs observed during embryogenesis. In 2023, Park et al. co‐cultured brain organoids with macrophages derived from the same iPSC line (iMACs). iMACs differentiated into cells exhibiting microglial phenotypes in organoids. Microglia delivered high levels of PLIN2^+^ lipid droplets, which were taken up by NPCs in the organoids, regulating their differentiation and enhancing axonogenesis.^[^ ^123^ ^]^ This work enhances the understanding of intercellular crosstalk during neural development and advances the design of neural organoids capable of simulating the immune microenvironment. Researchers have meticulously controlled the fusion ratio of NPCs and PMPs, generating organoids replicating the cellular composition in the human brain. In these organoids, microglia displayed phagocytic activity and synaptic pruning functions.^[^ ^124^ ^]^ Region‐specific organoids, including midbrain organoids and choroid plexus organoids (ChPOs), have been fused with macrophage precursor cells.^[^ ^121^, ^125^, ^126^, ^127^ ^]^ These models are valuable for studying neurodegenerative diseases and neuroinflammatory conditions. Several research teams have utilized erythromyeloid progenitors (EMPs), generated from the yolk sac, as precursor cells for fusion with neural organoids. Schafer et al. co‐cultured EMPs with cortical organoids, followed by xenotransplantation. This approach yielded vascularized immunocompetent human brain organoids (iHBOs), in which the microglia exhibited transcriptional profiles and behavior akin to those found in vivo. These microglia actively monitor the cerebral microenvironment, respond to local injuries, and react to systemic inflammatory signals.^[^ ^128^ ^]^ Fagerlund et al. confirmed that EMPs migrate to brain organoids, maturing into microglia‐like cells that interact with synapses, thereby promoting neuronal maturation.^[^ ^129^ ^]^ Organoid‐on‐a‐chip and multicellular 3D printing techniques have also been employed to establish organoid models for simulating neuroimmune interactions.^[^ ^130^, ^131^ ^]^

Microglia‐containing neural organoids serve as a powerful platform for studying viral infections and neural inflammation (Figure 4C), as they can replicate the cellular interactions between neurons and glial cells.^[^ ^121^, ^132^, ^133^ ^]^ Studies indicate that microglia are the only HIV target cells within brain organoids, revealing that microglial susceptibility is dependent on the co‐expression of the microglia‐specific markers and the CD4 and CCR5 HIV receptors.^[^ ^134^, ^135^ ^]^ Qiao et al. developed ChPOs containing microglia, demonstrating that microglia can effectively protect the epithelial barrier of HSV‐1‐infected ChPOs through the innate immune cyclic GMP‐AMP synthase (cGAS)‐STING pathway.^[^ ^136^ ^]^ Following infection with Zika virus (ZIKV) or SARS‐CoV‐2, microglia undergo dynamic changes that contribute to the excessive synaptic pruning and disrupt circuit integrity, ultimately leading to cognitive impairments.^[^ ^124^, ^137^ ^]^ Microglia play a significant role in the pathogenesis of various neurological diseases. Prenatal infections and activation of the maternal immune system can lead to neurodevelopmental disorders in the fetus, and the absence of microglia in conventional organoids complicates the simulation of prenatal risk factors affecting neural development. Alice et al. developed organoids containing microglia (COiMg), COiMg exhibited significant transcriptional and structural changes in response to interferon‐gamma (IFN‐γ) stimulation, displaying the expression of autism‐related genes.^[^ ^138^ ^]^ Researchers developed brain organoids containing microglia with SCN2A protein‐truncating mutations to simulate autism spectrum disorder (ASD). In this model, microglia exhibited increased elimination of post‐synapses, consistent with the phenotypes observed in animal models of the mutation.^[^ ^139^ ^]^ Brain organoids containing microglia have also been applied in modeling neurodegenerative disorders (Figure 4C). In neurodegenerative tissues, a subset of microglia known as “disease‐associated microglia” (DAM) has been reported. Takata et al. observed this subset in microglia‐containing cerebral organoids treated with Aβ protein.^[^ ^140^ ^]^ In the early stages of Alzheimer's disease (AD), the inflammatory response of microglia helps to clear amyloid‐beta (Aβ) and other neurotoxic metabolites; however, a prolonged inflammatory environment can lead to further neurodegeneration. Kuhn et al. established organoids derived from iPSCs with PSEN2 mutations, incorporating microglia to assess the dynamic immune environment in AD. They found that the reduction in synaptic density observed in this model could be prevented through the depletion of microglia.^[^ ^141^ ^]^

#### 3.2.2 Vascularized Neural Organoids

**Figure 5:**
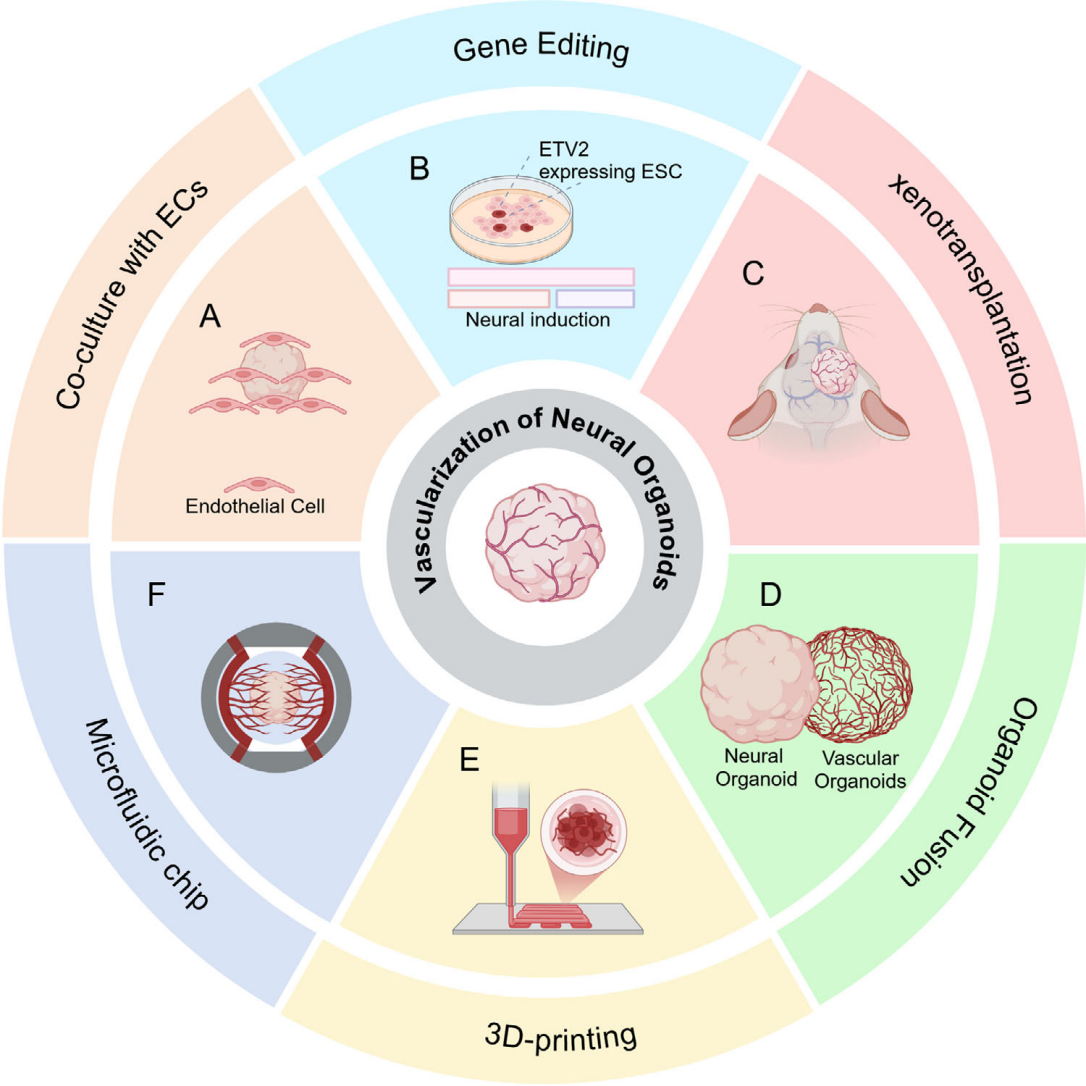
Methodologies for generating vascularized neural organoids. A) Vascularization of neural organoids by co‐culture with endothelial cells, B) expression of ETVs in hESCs, C) Xenotransplantation into rodents, D) Fusing with vascular organoids or mesodermal organoids, E) 3D‐printing with biomaterials and endothelial cells and a microfluidic chip. Created by Biorender.com.

In neural organoid cultures, the initial cell number typically ranges from 10^3^ to 10^4^.^[^ ^142^ ^]^ As NPCs proliferate, they can develop into dense neurospheres containing over 10^6^ cells. In the later stages of neural organoid development, the absence of vascularization leads to the formation of a necrotic core, which further causes spatial inconsistencies in neural development, disorganization of structure, and restricted size.^[^ ^125^ ^]^ Consequently, these organoids fail to accurately recapitulate the developmental features of the CNS. Furthermore, stimulation from endothelial cells is essential for the differentiation of NPCs.^[^ ^143^ ^]^ The integration of the vascular system also facilitates the establishment of blood–brain barrier (BBB)‐like structures and the synchronized development of microglia.^[^ ^144^ ^]^ Enhancing the permeability of the central region of neural organoids to improve neuronal maturity and diversity has become a major focus for many researches. Various strategies, including genetic engineering, assembloids, microfluidics, 3D bioprinting, and xenotransplantation, have been explored to promote vascularization within these tissue structures (**Figure** **5**).

Some studies have directly employed endothelial cell co‐culture to generate brain organoids or concentroids (NPC spheroid aggregates) containing vascular‐like structures (Figure 5A).^[^ ^92^, ^145^, ^146^ ^]^ While this method for vascular formation is straightforward and convenient, it may lead to issues such as the random distribution of endothelial cells within the organoids, as well as uncontrolled vascular location and density. With the ectopic expression of the endothelial gene ETV2, vascular‐like channels have been induced within organoids (vhCOs), leading to reduced cell death and enhanced neurogenesis (Figure 5B). vhCOs exhibit several characteristics of the BBB, including increased expression of tight junctions, nutrient transport proteins, and trans‐endothelial electrical resistance, while also supporting the formation of perfusable blood vessels after transplantation.^[^ ^82^ ^]^ Through xenotransplantation, brain organoids integrate with the vascular bed of the choroidal fissure in the cortex of rodents over ≈2 weeks (Figure 5C). This integration results in a rich distribution of blood vessels within the organoids, accompanied by cellular infiltration and synaptic signaling between the host and the organoid, effectively reduce apoptosis within the organoids and improve neural function.^[^ ^147^ ^]^ In addition to xenotransplantation, organoid fusion techniques, called “assembloids”, also provide an approach for the spontaneous infiltration of neurovascular interactions (Figure 5D). By assembling neural organoids with mesodermal organoids, models are generated in which neurons project toward the mesoderm, simulating the interactions between peripheral ganglia and vascular networks. This approach allows for the observation of NC cell differentiation and migration.^[^ ^148^ ^]^ The integration of brain organoids with vascular organoids (VOs) results in the formation of vascularized brain organoids.^[^ ^144^, ^149^ ^]^ These fused organoids not only exhibit functional BBB‐like structures but also demonstrate an increased abundance of neural cells and microglia. However, both xenotransplantation and organoid fusion may impact the self‐organizing architecture of neural organoids.

Tissue engineering serves as a powerful tool for shaping vascular structure and function. Functional biomaterials can act as vascular‐like scaffolds, providing nutrients to the central regions of neural organoids. When combined with technologies such as 3D printing and microfluidics, these techniques facilitate the development of vascular structures and functions (Figure 5E,F). Porous scaffold materials generated by two‐photon polymerization (TPP) 3D printing, which do not rely on endothelial cell support, serve as flow channel structures that inhibit the formation of necrotic cores within neural organoids.^[^ ^150^ ^]^ Researchers employed 3D printing to create high‐throughput, tunable, and reproducible scaffolds that support endothelial cell growth within channels and enable their migration into organoids.^[^ ^89^ ^]^ Another research utilized fluid dynamics to controllably mix biological materials, including cells, in a process known as “hydrodynamic focusing.” This application of biomaterials has resulted in the formation of ordered microtube‐like structures that effectively support the growth of neural‐like tissues.^[^ ^151^ ^]^ Salmon et.al designed a neurovascular organoid chip using 3D printing, enabling the self‐organization of pericytes and endothelial cells derived from iPSCs into a vascular system that co‐develops with neural organoids.^[^ ^152^ ^]^ At gestational weeks 6–7, a perineural vascular plexus (PNVP) forms, contributing to the early brain barrier along with various non‐neural cell types. Kaushik et.al cultured endothelial cells, NPCs, and microglia alongside primary pericytes (PCs) in a synthetic hydrogel, creating a microfluidic system that supports neural cell growth. This system serves as a predictive platform for developmental toxicity.^[^ ^153^ ^]^ Additionally, other studies have employed genetic engineering combined with tissue engineering techniques to create patterned neural tissues containing both neurons and endothelial cells, referred to as orthogonally induced differentiation (OID) of stem cells. This approach aims to generate programmable organoids to facilitate the application of engineered tissues in the biomedical field.^[^ ^154^ ^]^

The disruption of the BBB is a significant pathogenic factor in AD, with pericytes and endothelial cells being the primary components of the BBB. Recent reports also indicate that organoids enriched with choroidal epithelial cells can help establish models for BBB disruption in disease contexts.^[^ ^155^ ^]^ There is a correlation between COVID‐19 and AD, researchers developed assembloids by fusing cortical organoids with vascular organoids, which, upon infection with the SARS‐CoV‐2 virus, exhibit AD pathology, including the formation of β‐amyloid plaques. This provides a model in the plate for studying neuropathological changes induced by viral infection.^[^ ^156^ ^]^ iPSCs from ALS patients have been used to construct sensorimotor organoids, which not only contain neuroectodermal cell populations but also develop mesodermal cells, including microglia, blood vessels, and skeletal muscle. This enables comprehensive studies of neuromuscular junction (NMJ) dysfunctions associated with ALS.^[^ ^157^ ^]^ Researchers have integrated over seven different retinal cell types derived from human iPSCs to create a retinal chip that features vascular perfusion and recapitulates the side effects of drugs such as gentamicin in retinal pathologies.^[^ ^158^ ^]^

While neural organoids enriched with mesodermal cells have significantly contributed to the refinement of developmental and disease models in plates, the challenge of precisely adjusting the distribution and quantity of these cells to better reflect physiological and pathological characteristics remains an unresolved issue.

## 4 Applications of Neural Organoids

Neural Organoids are widely utilized in research to investigate the pathogenesis of neurological disorders, and act as an efficient platform for drug screening. Neural organoid transplantation can induce a bidirectional interaction between the graft and the host, offering a potential approach for neural repair.

### 4.1 Application of Brain Organoids in Disease Modeling and Neurodevelopmental Research

Brain organoids can model various neural disorders such as Alzheimer's disease (AD), Parkinson's disease (PD), epilepsy, allowing researchers to gain insights into the pathogenesis, pathophysiology, and alterations in signaling pathways associated with these disorders (**Table** **3**). Additionally, the generation process of neural organoids can mimic critical neurodevelopmental processes such as neurogenesis, subtype specification, and cell migration, it serves as an effective model for studying neurodevelopmental disorders. The application of brain organoids enables drug screening and evaluation, accelerating the innovative clinical therapies.

**Table 3:** Recent applications of brain organoids for modeling of neurological diseases.

Disease	Organoid used	Findings	Refs.
AD	Neural ectoderm organoids	Time dependent Aβ accumulation similar to AD pathology was found in Aftin‐5 induced neural ectoderm organoids, demonstrating that such organs can be used to identify factors in the environment that contribute to the occurrence of AD.	[161]
AD	Cortical organoids	Data indicated that tau increases with neuronal maturation in iPSC‐derived organoids similar with the developing fetal brain and forms a basis for research on regulatory mechanisms triggering the onset of tau gene transcription and translation.	[205]
AD	Cortical organoids	These findings enable modeling genetic AD in a human cellular context in a 3D cortical‐like tissue developed in vitro from patient‐specific stem cells.	[164]
AD	Retinal organoids	Familial AD retinal organoids exhibiting a significant increase in the Aβ 42/Aβ 40 ratio as well as phosphorylated Tau protein, characteristic of AD pathology.	[165]
AD	Brain organoids	The BOs carrying the BACE2 loss‐of‐function mutation (BACE2^G446R^) showed greater apoptosis and increased levels of Aβ oligomers compared to the control BOs, resembling with the AD‐associated phenotypes.	[167]
AD	Cortical organoids	The premature neurogenesis in fAD iPSCs harboring PSEN1 mutations was observed in cortical differentiation in 2D and cerebral organoid generation in 3D, respectively, which is partly driven by reduced Notch signaling.	[168]
AD	Brain organoids (Neuroectoderm)	Recapitulate AD pathologies in an age‐dependent manner in organoids derived from multiple fAD patients harboring APP duplication or PSEN1 mutation. Treatment of patient‐derived organoids with β‐ and γ‐secretase inhibitors significantly reduces amyloid and tau pathology.	[169]
AD	Cerebral organoids	APOE4 exacerbates tau pathology in both healthy subject‐derived and AD patient‐derived organoids. Cerebral organoids from AD patients are associated with an enhancement of stress granules and disrupted RNA metabolism. Isogenic conversion of APOE4 to APOE3 attenuates the APOE4‐related phenotypes in cerebral organoids from AD patients.	[171]
PD	Midbrain organoids	Use medical CFs as a novel scaffold for organ culture, with its porosity and stability in the cellular environment, addressed internal necrosis in organoids caused by long‐term cultivation.	[175]
PD	Midbrain organoids	They constructed a midbrain organoid with spatially organized groups of dopaminergic neurons. Detection of synaptic connections, electrophysiological activity, and myelination.	[176]
PD	Midbrain organoids	They described the robust generation of MOs with homogeneous distribution of mDA neurons, which exhibit mDA neuron‐specific cell death upon treatment with 1‐methyl‐4‐phenyl‐1,2,3,6‐tetrahydropyridine.	[177]
PD	Midbrain‐like simBOs	Established midbrain‐like simBOs from a PD disease patient (LRRK2^G2019S^)‐derived pNSCs, and found PFE‐360 could relieve the phenotype of PD in midbrain‐like simBOs.	[178]
PD	Midbrain organoids	Generated midbrain organoids carrying the LRRK2G2019S mutation, recapitulating the pathological features of patients with LRRK2‐associated sporadic PD, and revealed the functional role for TXNIP in the 3D environment of LRRK2‐associated PD.	[182]
PD	Midbrain organoids	Induced midbrain organoids lacking DJ1 activity, and found lysosomal protein hydrolysis is impaired, leading to AGE accumulation and increased phosphorylation of α‐syn.	[179]
PD	Midbrain organoids	By constructing PARK7 mutant midbrain organoids for drug screening optimization, they identified and validated a combination treatment strategy using the small molecules RECTAS and butyrate, which can restore patient‐derived fibroblast DJ1 protein and mitochondrial function, as well as rescue the loss of dopaminergic neurons.	[180]
PD	Midbrain organoids	Patient specific midbrain organoids show aggregation ofα‐syn and DA loss, and pathology in organoids is associated with a senescence‐like phenotype in astrocytes.	[181]
PD	NA	Statistical differences in the expression levels of LIM homeobox transcription factor alpha (early) and tyrosine hydroxylase (late) markers between organoids from PD patient and healthy volunteer.	[183]
Epilepsy	Cortical organoids	Uncovering the critical role of PCDH19 in human ventricular zone radial glial tissue and early cortical development.	[187]
DEE	Brain organoids	Discovered dramatic cellular and molecular CNS abnormalities, including neural population changes, cortical differentiation malfunctions, and Wnt pathway and DNA damage response impairment.	[188]
ASD	Cortical organoids	Cntnap2 regulated the differentiation of GABAergic neurons in early fetal cortical development. The anti‐epileptic drug retigabine effectively restores the number of GABAergic neurons in Cntnap2 KO cortical organoids, demonstrating the efficacy of Cntnap2 KO in treating ASD.	[190]
ASD	Forebrain organoids	Forebrain organoids generated from iPSCs of individuals with ASD carrying mutations in the CNTNAP2 gene typically exhibit clinical features of brain overgrowth, while Cntnap2 KO effectively inhibits organ overgrowth.	[191]

**AD,** Alzheimer's Disease; **AGEs,** Advanced Glycation End Products, **APOE,** Apolipoprotein E; **ASD,** Autism Spectrum Disorder; **BACE2**, Beta‐secretase 2; **BOs,** Brain Organoids; **CFs,** Carbon Fibers; **Cntnap2,** Contactin‐associated protein‐like 2; **DA**, Dopamine; **DEE,** Developmental and Epileptic Encephalopathy; **fAD,** familial AD; **iPSC**, induced Pluripotent Stem Cells; **LRRK2,** Leucine‐rich repeat kinase 2; **LRRK2^G2019S^ mutation**, Leucine‐rich repeat kinase 2 G2019S gene mutation; **mDA**, midbrain Dopaminergic; **MOs,** Midbrain Organoids; **PARK7,** Parkinson disease protein 7; **PCDH19,** Pathogenic calcium adhesion molecule‐19; **PD,** Parkinson's Disease; **pNSCs,** primitive Neural Stem Cells; **PSEN1,** Presenilin 1; **simBOs,** simplified Brain Organoids; **TXNIP,** Thioredoxin‐interacting protein.

#### 4.1.1 Alzheimer's Disease

AD is an age‐related neurodegenerative disorder characterized by synaptic changes, neuronal death, inflammation, and the accumulation of protein aggregates in the form of amyloid plaques (Aβ) and neurofibrillary tangles in the brain.^[^ ^159^ ^]^ While numerous animal and in vitro models have been developed for AD, there is a lack of an experimental approach that fully recapitulates the key disease features in the human brain. Brain organoids have emerged as a significant tool for studying the pathological characteristics of AD and for screening clinical drugs.

**Figure 6:**
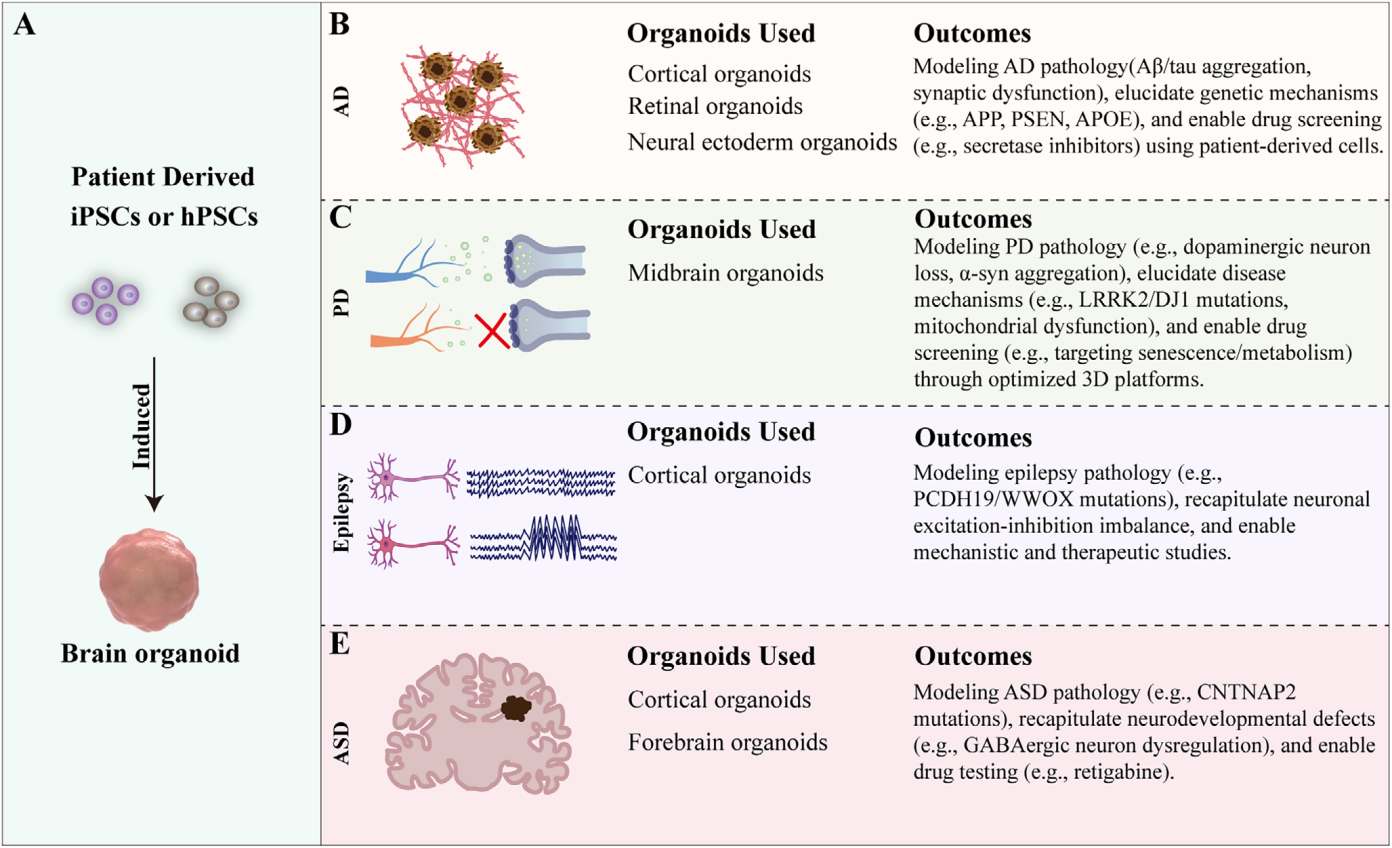
Application of brain organoids in neural disease modeling. (A) Schematic of brain organoids induced from patient derived iPSCs or hPSCs. Application of brain organoids in B) Alzheimer's Disease modeling, C) Parkinson's Disease modeling, D) Epilepsy modeling E) Autism spectrum disorder modeling. Created by Adobe Illustrator 2024.

Constructing brain organoids using patient‐derived cells is an effective means of modeling diseases, allowing for a deeper exploration of disease pathophysiology (**Figure** **6**A). The deposition of Aβ is a prominent feature in the pathological progression of AD.^[^ ^160^ ^]^ However, the physiological and pathological roles of amyloid proteins derived from amyloid precursor protein (APP), as well as their interactions with other proteins, are not fully established. Researchers have delved into the phenomenon of Aβ deposition by AD‐modeling brain organoids (Figure 6B). They observed time‐dependent Aβ deposition by Aftin‐5 treatment in human mini‐brains. Mini‐brains derived from wild‐type iPSCs exhibited responsiveness to compound induction, resulting in physiological shifts in Aβ concentrations, suggesting the model's utility in identifying environmental factors that may trigger sporadic AD onset.^[^ ^161^ ^]^ Tau protein hyperphosphorylation is another hallmark of AD, expressed throughout brain development, yet the timing and cell types in which this expression occurs, and its impact on disease states during fetal and neonatal periods, remain unclear.^[^ ^162^ ^]^ Researchers discovered that tau increases with the maturation of neurons in developing fetal brains and organoids by scRNA‐seq and RNA scope, laying the groundwork for future investigations into the regulatory mechanisms initiating tau gene transcription and translation. This potentially represents a therapeutic target for neurodegenerative tauopathies and developmental neurological disorders.^[^ ^163^ ^]^

Researchers have utilized iPSCs derived from patients with familial AD (fAD) to generate brain organoids. Over time, these organoids spontaneously exhibit pathological features akin to AD, including accumulations resembling Aβ and neurofibrillary tangles. These findings have enabled the modeling of genetic AD in vitro using cortical‐like 3D tissues derived from individual patient stem cells.^[^ ^164^ ^]^ Additionally, retinal organoids induced from hPSCs of fAD patients demonstrate a significant increase in the Aβ42/Aβ40 ratio and phosphorylated Tau protein. Transcriptomic analyses reveal pathway alterations associated with synaptic dysfunction, underscoring the retinal organoids' potential as a robust model for identifying early retinal changes linked to AD.^[^ ^165^ ^]^

Generation of brain organoids based on disease risk genes is another strategy for studying disease mechanisms. β‐secretase 2 (BACE2), a homolog of the promising AD therapeutic target BACE1, is poorly understood in terms of expression and functional roles in the CNS.^[^ ^166^ ^]^ By developing BACE2 loss‐of‐function mutation (BACE2^G446R^) brain organoids from hPSCs, researchers found that the BACE2 functional mutation led to increased neuronal apoptosis and elevated levels of Aβ oligomers, resembling AD‐associated phenotypes. These disease phenotypes were rescued by removing the APP protein in the BACE2 mutation organoids, revealing the protective role of BACE2 in preventing apoptosis induced by Aβ deposition.^[^ ^167^ ^]^ Mutations in Presenilin 1 (PSEN1) or Presenilin 2 (PSEN2) lead to fAD. Researchers utilized iPSCs derived from fAD patients with PSEN1 mutations to develop brain organoids, revealing a trend of reduced numbers of newborn neurons in fAD cases, manifesting premature aging phenotypes.^[^ ^168^ ^]^ In another study using iPSCs derived from patients with fAD to construct brain organoids, treatment with β‐ and γ‐secretase inhibitors significantly reduced amyloid and tau pathology in patient‐derived brain organoids.^[^ ^169^ ^]^ The ε4 allele of *APOE* gene (APOE4) is the most related genetic risk factor for late‐onset AD.^[^ ^170^ ^]^ To explore its potential mechanisms, researchers developed brain organoid models using iPSCs from individuals with normal cognition or AD dementia carrying the APOE ε3/ε3 or ε4/ε4 genotypes. Transcriptomic sequencing revealed enhanced stress granules and disrupted RNA metabolism in brain organoids derived from AD patients.^[^ ^171^ ^]^ Pitrilysin metallopeptidase 1 (PITRM1) is a mitochondrial protease involved in mitochondrial precursor processing and degradation. Mutations of PITRM1 lead to a syndrome characterized by cerebellar ataxia, psychiatric episodes, compulsive behaviors, and cognitive decline. Using cortical neurons and brain organoids generated from PITRM1 gene knockout iPSCs, Pérez et al. revealed a mechanistic link between mitochondrial function and common neurodegenerative proteinopathies.^[^ ^172^ ^]^ Starting from different aspects of AD pathology, these efforts construct brain organoid to deeply analyze its pathological mechanisms, providing a solid theoretical basis for clinical treatment.

#### 4.1.2 Parkinson's Disease

PD is a common neurodegenerative disorder characterized by the loss of A9‐specific DA neurons in the substantia nigra pars compacta (SNpc) of the midbrain.^[^ ^173^ ^]^ Despite extensive research, the etiology of PD remains unresolved, and there remains no cure for PD. One reason for this dilemma is the lack of a disease model that faithfully replicates the characteristics of human PD. The advent of brain organoid has made it possible to establish PD models using patient‐derived cells.^[^ ^174^ ^]^ Researchers have overcome challenges in organoid construction through technological improvements to better study the pathological mechanisms of PD. For instance, the use of medical carbon fibers (CFs) as a novel scaffold for midbrain organoid generation, with its porosity and stability in the cellular environment, has addressed necrotic nucleus in organoids.^[^ ^175^ ^]^ The precise control of the structure of dopaminergic neurons represents another challenge in the construction of midbrain organoid. In order to address this issue, Monzel et al. constructed human midbrain organoids with spatially ordered groups of DA neurons and observed synaptic connections and electrophysiological activity, providing a more realistic simulation of midbrain structure, with significant potential for disease modeling and clinical therapeutic strategy development.^[^ ^176^ ^]^ The low efficiency of midbrain organoid generation and the relatively immature and heterogeneous structure hinder the translation of these models from the laboratory to the clinic. Kwak et al. have generated organoids with evenly distributed mDA neurons, showing mDA neuron‐specific cell death upon treatment with 1‐methyl‐4‐phenyl‐1,2,3,6‐tetrahydropyridine, proving the utility of such organoids as a model system for studying the in vivo pathology of PD.^[^ ^177^ ^]^ Heterogeneity and long‐term differentiation (over 2 months) also impede the application of organoids. In response to this issue, researchers have established simplified brain organoids (simBOs) composed of mature neurons and astroglial cells from hPSC‐derived primitive neural stem cells (pNSCs). simBOs can be rapidly generated in 2 weeks and exhibit more homogeneous properties.^[^ ^178^ ^]^ The works aforementioned make it possible to construct better midbrain organoids to simulate the pathological mechanisms of Parkinson's disease.

Parfitt et al. induced midbrain organoids lacking DJ1 activity from iPSCs to investigate how advanced glycation end products (AGEs) promote the development of early‐onset PD caused by loss of DJ1 protein deglycase function. In midbrain organoids lacking DJ1 activity, lysosomal protein hydrolysis is impaired, leading to AGE accumulation and increased phosphorylation of alpha‐synuclein (α‐syn). Loss of DJ1 diminishes the ability of astrocytes to provide metabolic support, triggering an inflammatory phenotype that results in neurodegeneration.^[^ ^179^ ^]^ In another study, researchers identified a U1‐dependent splicing defect in an in vitro model of PD associated with Parkinson disease protein 7 (PARK7), leading to a sharp reduction in DJ1 protein levels and subsequent mitochondrial dysfunction. By constructing mutant midbrain organoids for drug screening optimization, they identified and validated a combination treatment strategy using the small molecules RECTAS and butyrate, which can restore patient‐derived fibroblast DJ1 protein and mitochondrial function, as well as rescue the loss of DA neurons in mutant midbrain organoids.^[^ ^180^ ^]^

The DA neuron loss in the substantia nigra pars compacta of the midbrain is a hallmark feature of PD. Researchers have replicated key pathological features of PD using patient‐derived human midbrain organoids and identified a link between these pathological markers and an increase in the senescent cell phenotype in astrocytes, demonstrating the role of pathological α‐syn in inducing astrocyte senescence (Figure 6C).^[^ ^181^ ^]^ Midbrain organoids carrying the LRRK2^G2019S^ mutation (Leucine‐rich repeat kinase 2 G2019S gene mutation) were generated to recapitulate the pathological features of patients with LRRK2‐associated sporadic PD. Analysis of the protein–protein interaction network in LRRK2 mutant organoids revealed a functional role for thioredoxin‐interacting protein (TXNIP) in the 3D environment of LRRK2‐associated PD, providing a theoretical basis for modeling sporadic PD based on organoids and advancing PD therapeutics.^[^ ^182^ ^]^ Chlebanowska et al. generated large‐scale, multicellular organoids from iPSCs derived from peripheral blood monocytes of healthy volunteers and idiopathic PD patients. They identified statistical differences in the expression levels of the early LIM homeobox transcription factor α and the late tyrosine hydroxylase markers between PD patients and healthy individuals.^[^ ^183^ ^]^ These Parkinson's disease pathological researches conducted through midbrain organoid models has provided us with a clearer understanding of the pathogenesis and progression of PD.

#### 4.1.3 Epilepsy

Epilepsy is a neurological disorder commonly occurring in childhood, characterized by an imbalance in neuronal electrical activity associated with dysregulation between neuronal excitation and inhibition.^[^ ^184^ ^]^ Causes of epilepsy include stroke, infections, brain injuries, genetic factors, or other alterations in brain structure, with many idiopathic cases. Animal models and in vitro experiments aid in understanding epilepsy, yet improved models are needed to delve deeper into the pathogenesis of epilepsy and develop therapeutic targets. Brain organoids recapitulate human brain features absent in animal models, enabling more in‐depth studies of epilepsy (Figure 6D).^[^ ^185^, ^186^ ^]^

Pathogenic calcium adhesion molecule‐19 (PCDH19)‐related clustering epilepsy (PCE) is a developmental epileptic encephalopathy resulting from loss‐of‐function mutations in the PCDH19 gene on the X chromosome. Researchers generated human cortical organoids using homologous female hESCs with mixed wild type (WT) or homozygous PCDH19 knockout (KO), uncovering the critical role of PCDH19 in human ventricular zone radial glial tissue and early cortical development.^[^ ^187^ ^]^ Additionally, a study utilized hESCs edited for the tumor suppressor gene WW domain‐containing oxidoreductase (WWOX) and patient‐derived iPSCs to establish brain organoids modeling developmental and epileptic encephalopathy (DEE) in vitro. This in vitro organoid model revealed significant abnormalities in central nervous system cells and molecular features, including changes in neuronal proportions, cortical differentiation dysfunction, and impairments in Wnt signaling pathways and DNA damage responses. Subsequent experimental findings suggest that ectopic WWOX expression holds promise in rescuing these phenotypes. These studies demonstrate the immense potential of brain organoids for modeling pediatric epileptic encephalopathies and employing them as platforms for drug screening.^[^ ^188^ ^]^

#### 4.1.4 Autism Spectrum Disorders

ASD refer to a range of neurodevelopmental conditions characterized by challenges with social skills, repetitive behaviors, communication difficulties, and often unique strengths and differences.^[^ ^189^ ^]^ Individuals with ASD can have a wide variety of symptoms and levels of impairment, leading to the term “spectrum”. ASD includes conditions such as autism, Asperger syndrome, and pervasive developmental disorder not otherwise specified (PDD‐NOS).

Variants of Contactin‐associated protein‐like 2 (Cntnap2), a member of the neurexin family of proteins, function as cell adhesion molecules. Loss of Cntnap2 function results in ASD in humans and mice. However, the cellular‐level functional impacts of these mutations during fetal development remain unclear. Utilizing Cntnap2 KO mice iPSCs‐derived mouse cortical organoids (mCOs), it was found that the cell adhesion molecule Cntnap2 plays a crucial role in regulating the differentiation of GABAergic neurons in early fetal cortical development. The anti‐epileptic drug retigabine effectively restores the number of GABAergic neurons in KO mCOs, demonstrating the efficacy of Cntnap2 KO mCOs in treating ASD.^[^ ^190^ ^]^ Forebrain organoids generated from iPSCs of individuals with ASD carrying mutations in the Cntnap2 gene typically exhibit clinical features of brain overgrowth, while Cntnap2 KO effectively inhibits the overgrowth. These studies highlight the opportunity provided by organoid models with Cntnap2 edition for the development of new therapeutic strategies for ASD.^[^ ^191^ ^]^ Furthermore, brain organoids derived from iPSCs of healthy controls and patients with primary progressive multiple sclerosis (PPMS), secondary progressive MS (SPMS), and relapsing‐remitting MS (RRMS) revealed an association between stem cell dysregulation in the stem cell pool and reduced expression of the cell cycle inhibitor p21. The genetic background of patients can directly alter stem cell function, providing new insights into inherent cellular dysregulation in MS and identifying the p21 pathway as a novel potential target for MS therapeutic strategies.^[^ ^192^ ^]^

#### 4.1.5 Other Disease Modeling by Brain Organoids

In addition to serving as platforms for establishing various models of neurological disorders, brain organoids represent valuable tools for assessing neurotoxicity. The human brain is a complex and intricately organized organ, susceptible to the influence of exogenous chemicals such as pollutants, drugs, and industrial substances, which may impact brain development or function, ultimately leading to neurological diseases. Brain organoids with self‐assembled 3D structures offer opportunities for human neurotoxicity assessments.^[^ ^193^ ^]^ Dolutegravir (DTG), an antiretroviral medicine, has been reported to increase the risk of neural tube defects (NTDs) in newborns when taken by pregnant women. Caiaffa et al. employed stem cell‐derived brain organoids to explore the potential mechanisms underlying DTG‐induced NTDs.^[^ ^194^ ^]^ Brüll et al. constructed 3D‐cultured human dopaminergic neurons and further integrated oligodendrocytes and astrocytes to form the organoid. This organoid, comprised of three cell types with customizable proportions, enables the quantification of toxicant effects on the organoid in a high‐throughput manner, paving the way for new avenues of research into the pathophysiology and toxicology of the human CNS.^[^ ^195^ ^]^ Another study combined the unguided method of brain organoid culture with forebrain organoid culture techniques to recapitulate the spatiotemporal features of early human brain development. This improved organoid platform, characterized by convenience and controllability, was utilized for neurotoxicity assessment of the environmental toxin cadmium, represents a powerful tool for research in neurodevelopment, neurological disorders, and neurotoxicology.^[^ ^196^ ^]^

#### 4.1.6 The Application of Brain Organoids in Deciphering Neural Developmental

Brain organoid technology, by mimicking the spatiotemporal features and cellular diversity of human brain development, has become a cornerstone tool for deciphering neural developmental mechanisms and disease pathology. Cutting‐edge research has revolutionized organoid culture paradigms through 3D bioprinting—the scaffold system developed not only precisely regulates the mechanical microenvironment (with stiffness matching human brain tissue) but also pioneers endothelialized channel co‐culture, establishing a new model for neuro‐vascular interaction studies.^[^ ^89^ ^]^ Cross‐species comparative research utilizing single‐cell multi‐omics technologies revealed that human cortical progenitor cells exhibit a unique “slow development” pattern, with delayed gene expression timing and distinct chromatin accessibility dynamics compared to chimpanzees, potentially explaining the evolutionary basis of human brain complexity.^[^ ^197^, ^198^ ^]^ Notably, organoid morphological characteristics have been proven to directly influence cell fate determination—spatial transcriptomics demonstrated that structurally abnormal organoids cause spatiotemporal disorganization of neuroepithelial cells, suggesting developing brain cells require precise physical microenvironment cues to maintain normal differentiation trajectories. In disease modeling,^[^ ^199^ ^]^ Cheroni et al. established a cross‐platform transcriptomic benchmarking framework revealing heterochronic differentiation features between organoids and fetal cortex under different culture protocols,^[^ ^200^ ^]^ while CHOOSE system established by Li et al. identified through high‐throughput genetic perturbation screening that autism risk genes (e.g., ARID1B) disrupt ventral progenitor‐to‐interneuron conversion by impairing BAF complex function.^[^ ^201^ ^]^ These breakthroughs collectively propel brain organoids from basic research toward precision medicine applications, providing a systematic platform for uncovering molecular mechanisms of neurodevelopmental disorders and developing targeted intervention strategies.

### 4.2 Applications of Spinal Cord Organoid in Disease Modeling

Research on SCOs has greatly facilitated the elucidation of the pathophysiology of SCI and NT developmental disorders, as well as drug screening efforts.

#### 4.2.1 Amyotrophic Lateral Sclerosis

Amyotrophic lateral sclerosis (ALS), also known as Lou Gehrig's disease, is a progressive neurodegenerative disorder that affects nerve cells in the brain and spinal cord. This condition leads to the gradual degeneration and loss of motor neurons, which are responsible for controlling voluntary muscle movements. As ALS progresses, individuals may experience muscle weakness, twitching, and eventually muscle atrophy. This can result in difficulties with speaking, swallowing, and eventually breathing. The cause of ALS is not fully understood, but it is believed to involve a combination of genetic and environmental factors. There is currently no cure for ALS, and treatment focuses on managing symptoms, providing supportive care, and improving quality of life for individuals with the disease.^[^ ^211^, ^212^ ^]^ Guo et al. utilized C9orf72‐silenced (C9) iPSCs to generate SCOs mimicking ALS pathology, demonstrating similar differentiation abilities between WT and edited iPSCs. RNA sequencing revealed upregulated pro‐inflammatory factors in C9‐iPSC‐derived cells.^[^ ^213^ ^]^ Additionally, a trunk neural muscle organoid system derived from ALS iPSCs replicated ALS peripheral defects. ALS neural muscle organoids exhibited RNA foci, dipeptide repeat proteins, and responded positively to treatment with the protein unfolding stress inhibitor GSK2606414, showing potential for drug testing in ALS research.^[^ ^214^ ^]^

#### 4.2.2 Spinal Muscular Atrophy

Spinal Muscular Atrophy (SMA) is a neuro‐muscular disease caused by mutations in the SMN1 gene, leading to a significant reduction in Survival Motor Neuron (SMN) protein levels.^[^ ^215^, ^216^ ^]^ While SMN is widely expressed throughout the body, spinal motor neurons are among the most severely affected cell types. Researchers have developed a spinal cord organoid model for SMA, demonstrating marked degeneration of motor neurons within it, which can be prevented with a small molecule inhibitor of CDK4/6.^[^ ^217^ ^]^ Another study explores the hypothesis of whether developmental defects contribute to postnatal neuronal death in SMA. Utilizing iPSC models and SCOs systems, findings show that SMA SCO exhibits abnormal morphological development, reduced expression of neuronal precursor markers, and accelerated expression of midline precursor and muscle cell markers. These results suggest that early neurodevelopmental abnormalities may lead to subsequent motor neuron degeneration, emphasizing that interventions to increase SMN postnatally may not fully reverse all pathological features of SMA.^[^ ^104^ ^]^

#### 4.2.3 Neural Tube Defects

The neural tube, the precursor of the entire CNS in vertebrates, forms through a conserved early developmental process known as neurulation. Disruptions in neurulation, caused by genetic or environmental factors, can lead to NTDs, the most common congenital malformations.^[^ ^218^, ^219^ ^]^ Recent research has introduced SCOs that replicate aspects of human neural tube formation and have shown that valproic acid (VPA) treatment in these organoid models may induce neural tube formation defects.^[^ ^220^ ^]^ Additionally, an advanced 3D culture system has been reported for generating human SCO‐like structures, which exhibit features resembling neural tube formation, including tubular morphogenesis, differentiation into major spinal cord neurons and glial cells, and mature synaptic functional activity.^[^ ^101^, ^221^ ^]^ These studies offer valuable biological resources for dissecting the molecular pathways governing previously unknown human neural tube formation processes.

#### 4.2.4 Pain

Neuropathic pain is a chronic condition often treated with long‐term opioid medications. However, prolonged use of opioids can lead to tolerance and/or hyperalgesia, diminishing their therapeutic efficacy.^[^ ^222^, ^223^ ^]^ Understanding the mechanisms behind opioid‐induced tolerance and hyperalgesia in humans has been challenging due to limitations in current models that fail to fully replicate human pathology. To address this gap, a human spinal cord micro physiological system (MPS) has been developed, integrating on‐demand neural activity sensing to simulate human pain perception and opioid‐induced tolerance.^[^ ^224^ ^]^ In parallel, a study introduces a human SCO‐chip device designed to mimic the biology and electrophysiology of human nociceptive neurons and spinal cord dorsal horn interneurons in the pain perception circuit. These advancements offer valuable tools for investigating the mechanisms of neuropathic pain, opioid tolerance, and hyperalgesia in a more human‐relevant context. By combining the MPS and organ‐chip technologies, researchers now have innovative platforms to explore neuropathic pain pathophysiology and to advance the discovery and validation of novel therapeutic approaches for pain management.^[^ ^225^ ^]^

#### 4.2.5 Other Diseases Modeling by SCOs

Research using SCOs has significantly advanced the study of neurological disorders by focusing on fetal spinal cord ischemia, mitochondrial DNA mutations such as those in MELAS, and the role of the Hedgehog (HH) pathway in neural development. A study established a screening system using SCOs to explore drug effects on fetal spinal cord ischemia, highlighting the utility of necrosis‐free human SCOs (nf‐hSCOs) for large‐scale screening.^[^ ^226^ ^]^ Another investigation delved into motor neuron pathology in mitochondrial diseases like MELAS using SCOs, revealing the involvement of heightened Notch signaling and the potential of the gamma‐secretase inhibitor DAPT in reversing neurodevelopmental defects.^[^ ^102^ ^]^ Additionally, SCOs were employed to quantitatively study HH pathway activity, showcasing their effectiveness in assessing HH signaling and offering insights into genetic and chemical contributions to pathway regulation. These studies collectively demonstrate the versatility and significance of SCOs in unraveling the complexities of neurological disorders at a cellular level.^[^ ^227^ ^]^

### 4.3 Transplantation of neural organoids

Researchers utilized intracerebral xenotransplantation methods to achieve vascularization of brain organoids and robust neuronal differentiation, providing comprehensive characterization of cellular fate changes within the transplanted organoids.^[^ ^147^, ^202^, ^203^, ^204^, ^205^ ^]^ These works provide a chance for organoid transplantation for neural repair.

Cell transplantation can reconstruct lost neural circuits in the cerebral cortex after injury, with brain organoid transplantation being a more effective therapeutic approach. However, due to the developmental changes in the cellular composition of organoids, it is currently unclear at which developmental stage of the organoid is most suitable for reconstructing the corticospinal tract. Kitahara et al. transplanted hESC‐derived brain organoids (differentiated for 6 or 10 weeks) into the cerebral cortex of mice, and found that brain organoids differentiated for 10 weeks exhibited significant axonal growth in the primate brain. These findings provide a basis for brain injury and stroke organoid transplantation therapy.^[^ ^204^ ^]^

Several studies have shown promising applications of brain organoid transplantation in the treatment of neurological disorders, demonstrating prospects in the neural repair after strokes and traumatic brain injuries (TBI). Transplanting brain organoids after SCI significantly promotes axonal regeneration in animals and achieves higher Basso Beattie Bresnahan scores compared to collagen gel transplantation.^[^ ^206^ ^]^ Cao et al. transplanted hPSC‐derived human brain organoids into the peri‐infarct core and peri‐infarct boundary of stroke NOD‐SCID mice, where the transplanted organoids survived well in the infarct core. The transplanted organoids differentiated into target neurons, repaired the infarcted tissue, extended axons to distal brain target areas, integrated into the host neural circuitry, thereby improving sensory‐motor deficits in stroke mice.^[^ ^207^ ^]^ Another study found that transplanting brain organoids 6 or 24 h after middle cerebral artery occlusion (MCAO) in a rat stroke model significantly reduced brain infarct volume and improved neurological function. The transplanted brain organoids promoted region‐specific reconstruction of the motor cortex area, established synaptic connections with the host brain through in situ differentiation and cell replacement. Additionally, brain organoid transplantation facilitated exogenous neurogenesis in the cortex surrounding the transplantation site and endogenous neurogenesis in the hippocampus and periventricular regions.^[^ ^208^ ^]^ Similarly, transplanting brain organoids differentiated for 55 and 85 days into the damaged motor cortex after TBI yielded similar therapeutic effects.^[^ ^209^ ^]^ Bao et al. transplanted hESC‐derived brain organoids into the brain injury site of TBI‐immunocompromised (SCID) mice, leading to vascularization post‐transplantation and reduced glial scarring in mice brains, indicating promotion of neural repair. The spatial learning and memory abilities of the mice also improved.^[^ ^210^ ^]^ These studies highlight the potential application of brain organoid transplantation in treating CNS injury diseases, providing preclinical evidence for the effective intervention of brain organoid transplantation in the treatment of neural injury.

SCOs are also directly employed for therapeutic purposes. For instance, SCOs can be transplanted into damaged areas of patients to aid in the repair or replacement of injured tissues or organs. This approach can be applied to treat a range of conditions and injuries, such as SCI, neurodegenerative diseases, and more. By utilizing SCOs for transplantation therapy, tissue regeneration and functional recovery can be promoted, offering patients hope and the potential to enhance their quality of life.

Researchers explored the direct reprogramming of human astrocytes into neurons or oligodendrocyte precursor cells, proposing a method to create specific microenvironments that could enhance SCI treatment.^[^ ^10^ ^]^ Another study utilized decellularized optic nerve (DON) as a scaffold, combined with oligodendrocyte precursor cells overexpressing neurotrophin‐3 (NT‐3), to develop white matter‐like tissue (WMLT). This approach led to significant improvements in motor function in a SCI model, overcoming challenges related to targeted axonal regeneration and myelination.^[^ ^228^ ^]^ Lai et al. introduced the construction of SCLT, emulating the white and gray matter composition of the spinal cord. By integrating WMLT and gray matter‐like tissue (GMLT) modules, targeted repair of damaged spinal cords was achieved. Transplanting SCLT into a rat SCI model resulted in notable restoration of motor function, increased remyelination in the WMLT region, enhanced innervation in the GMLT region, and supported neural signal conduction.^[^ ^229^ ^]^ These studies collectively underscore the potential of using tissue‐engineered SCOs as a novel strategy for treating SCI, offering valuable insights for advancing research in neural regeneration and repair.

Neural organoid transplantation as an emerging regenerative medicine technology demonstrates significant potential for repairing brain damage and restoring neurological functions. However, it faces multiple challenges: developmental stage limitations (only simulating fetal brain tissue, unable to replicate adult brain complexity), incomplete cell types and functions (insufficient differentiation of specific neurons/glia affecting host integration), lack of vascularization (impairing nutrient metabolism and blood–brain barrier function, restricting drug screening applications), and immunological evaluation gaps (inability to simulate post‐transplantation immune responses). Additionally, ethical concerns arise from the use of human pluripotent stem cells, including embryonic stem cell controversies and clinical compliance risks. These technical bottlenecks and ethical considerations collectively constitute critical barriers that must be addressed for clinical translation of brain organoid transplantation (**Table** **4**).

**Table 4:** Recent researches of neural organoids transplantation and main outcomes.

Organoid	Transplantation purpose	Effects or research conclusions	Refs.
Brain organoids (differentiated for 6 or 10 weeks)	Reconstruct neural circuits in the mouse cerebral cortex after injury	10‐week differentiated organoids exhibited extensive axonal growth into the host brain, forming synaptic connections with host neurons.	[204]
Brain organoids	Treatment of SCI	Axonal regeneration was accompanied by functional recovery (e.g., improved locomotor scores). Transplanted organoids integrated with host tissue and reduced glial scar formation	[206]
Brain organoids	Treatment of stroke in NOD‐SCID mice	Organoids survived in the infarct core and differentiated into glutamatergic/GABAergic neurons. Axons extended to distal brain regions (e.g., contralateral cortex and thalamus), restoring neural circuitry.	[207]
Brain organoids	Treatment of rat stroke model	Reduced infarct volume by 30–40% compared to controls. Promoted endogenous neurogenesis in the hippocampus and periventricular regions. Synaptic integration with host neurons confirmed by electrophysiology	[208]
Brain organoids (differentiated for 55 and 85 days)	Treatment of TBI	Both differentiation stages showed similar therapeutic efficacy. Transplanted organoids reduced inflammation and enhanced neurotrophic factor secretion.	[209]
Brain organoids	Treatment of TBI in immunocompromised mice	Vascularization of organoids occurred within 2 weeks post‐transplantation. Improved spatial memory (Morris water maze performance) correlated with reduced astrogliosis.	[210]
Spinal cord organoids	Treatment of SCI	Reprogrammed astrocytes contributed to remyelination. NT‐3‐overexpressing oligodendrocyte precursors enhanced axonal regeneration through DON scaffolds.	[10, 228]
White matter‐like tissue (WMLT)	Treatment of SCI	WMLT restored corticospinal tract integrity, confirmed by anterograde tracing. Myelination of regenerated axons improved motor‐evoked potentials	[228]
Spinal cord‐like tissue (SCLT)	Treatment of SCI	Bimodal repair: WMLT module supported axonal regrowth, while GMLT module restored synaptic connectivity. Electrophysiological recordings confirmed functional neural relay formation.	[229]

**DON,** Decellularized Optic Nerve; **GMLT,** Gray Matter‐like Tissue; **SCI,** Spinal Cord Injury; **SCLT,** Spinal Cord‐like Tissue; **TBI,** Traumatic brain injury; **WMLT,** White Matter‐like Tissue.

### 4.4 Applications of Neural Organoid in Drug Screening

Brain organoid technology has demonstrated immense potential in the field of drug screening, offering novel approaches to overcome the limitations of traditional animal models. Studies have shown that stem cell‐based organoids can more accurately simulate human organ development and disease characteristics.^[^ ^230^ ^]^ Given the selective permeability of drugs to the brain, damage or dysfunction of the BBB in various neurodegenerative diseases exacerbates pathological progression. Consequently, the BBB has emerged as a critical structural target for NDD therapeutic development and a potential primary site of action for brain‐penetrating drugs. Significant breakthroughs have been achieved in the development of BBB organoid models. Researchers have successfully constructed a fully human iPSC‐derived BBB‐on‐a‐chip model, which incorporates brain microvascular endothelial‐like cells, astrocytes, and neurons. This model accurately predicts drug permeability across the BBB and provides a platform for personalized medicine.^[^ ^231^ ^]^ Additionally, microfluidic BBB models, by mimicking in vivo BBB properties, have achieved stable cultures for up to 10 days, with transendothelial electrical resistance values reaching physiologically relevant levels, thereby offering a reliable tool for drug permeability studies.^[^ ^232^ ^]^ Notably, the development of choroid plexus organoids has further enriched the brain organoid system. These organoids not only secrete cerebrospinal fluid‐like liquid but also exhibit selective barrier functionality, enabling the prediction of central nervous system permeability for new compounds.^[^ ^72^ ^]^

In specific applications to neurological diseases, researchers have developed a cross‐platform screening workflow for cortical organoids using serum‐free embryoid bodies derived from iPSCs. This workflow integrates high‐content imaging, multi‐electrode arrays, and single‐cell calcium imaging, significantly enhancing experimental reproducibility and screening efficiency. This approach not only addresses the variability issues inherent in traditional 3D cultures but also provides a high‐throughput starting point for phenotypic drug screening.^[^ ^233^ ^]^ In mitochondrial disease research, brain organoids serve as a powerful tool for modeling human diseases and drug screening, owing to their ability to recapitulate brain development and function, thereby offering a potential platform for personalized medicine.^[^ ^234^ ^]^ In PD research, 3D brain organoid models were employed to evaluate the potential of the antiviral drug Tilorone in suppressing the propagation of α‐synuclein pathology. Results demonstrated that Tilorone significantly reduced the uptake and phosphorylation levels of pathogenic α‐syn fibrils and alleviated apoptosis.^[^ ^235^ ^]^ Furthermore, the development of a novel human spinal cord microphysiological system (MPS) has provided a new tool for studying opioid‐induced tolerance and hyperalgesia. This system integrates plug‐and‐play neural activity sensing technology to simulate human nociception and opioid‐induced tolerance, showcasing significant translational potential.^[^ ^224^ ^]^ In neural tube defect research, SCOs generated via a 3D culture system were successfully used to screen the effects of antiepileptic drugs on NTDs, validating the feasibility of this model for disease modeling and drug screening.^[^ ^101^ ^]^ Lastly, motor neuron organoid models have excelled in studies of oxidative stress‐induced axonal degeneration, providing a high‐throughput screening platform for evaluating cytoskeleton‐modulating drugs that promote axonal regeneration.^[^ ^236^ ^]^ These innovative models overcome the limitations of traditional animal models, such as interspecies differences and systemic complexity, providing an in vitro platform that more closely resembles human physiology for mechanistic studies and drug development in neurodegenerative diseases. With the integration of organoid and organ‐on‐a‐chip technologies, future advancements may enable the simulation of multi‐organ interactions, further enhancing the efficiency and accuracy of drug screening.

In addition to clinical drug screening, brain organoids have demonstrated unique advantages in evaluating the neurotoxicity of environmental chemical factors. Recent advancements in brain organoid technology have significantly enhanced the evaluation of neurotoxicity, offering platforms that more accurately recapitulate human brain development and pathology compared to traditional models. For instance, human iPSC‐derived brain organoids have been employed to assess the neurotoxic mechanisms of vincristine, a chemotherapeutic agent. Studies revealed dose‐dependent neurotoxicity, including reduced neuronal and astrocyte populations, impaired microtubule‐associated proteins (e.g., tubulin and fibronectin), and downregulated MMP10 activity, highlighting the utility of organoids in quantifying drug‐induced neurotoxicity.^[^ ^237^ ^]^ Similarly, improved forebrain organoid systems, cultured with transient Matrigel embedding, have demonstrated robust reproducibility in modeling early human brain development and neurotoxic responses. For example, cadmium exposure in these organoids caused severe developmental disruptions, such as cortical cell death and premature differentiation, underscoring their value in environmental toxicology.^[^ ^196^ ^]^ Innovative 2.5D cultures of LUHMES dopaminergic neurons further expanded neurotoxicity testing capabilities, enabling high‐throughput quantification of toxic effects from compounds like MPP+ and erastin. The integration of astrocytes or microglia into these systems revealed neuroprotective roles, such as astrocyte‐mediated mitigation of proteasome inhibitor toxicity.^[^ ^195^ ^]^ Additionally, brain organoids have elucidated the neurotoxic mechanisms of dolutegravir (DTG), an antiretroviral drug linked to neural tube defects. DTG exposure altered folate receptor (FOLR1) expression and disrupted genes critical for neurogenesis, while biomechanical analyses revealed increased surface stiffness and reduced internal organoid hardness.^[^ ^194^ ^]^ Collectively, these studies underscore the versatility of brain organoids in neurotoxicity assessment, bridging gaps between developmental sensitivity, drug‐induced toxicity, and environmental exposures. Their ability to model human‐specific responses and integrate multicellular interactions positions them as indispensable tools for advancing neurotoxicological research and drug safety evaluations.

Organoids hold significant promise for drug screening by modeling human tissue organization and disease phenotypes. However, current applications face several limitations: low generation efficiency restricts screening scale, while variability in quality compromises phenotypic reliability. Systematically, most models fail to replicate in vivo drug interactions with multi‐tissue systems, particularly lacking integration of critical physiological barriers like the BBB. Technical challenges, such as inadequate vascularization and circulatory system modeling, further limit physiological relevance, collectively reducing screening efficiency and clinical translatability. Future advancements should focus on optimizing generation protocols to enhance efficiency and consistency, integrating multi‐tissue interactions and physiological barriers, overcoming vascularization bottlenecks, and leveraging high‐throughput technologies to improve precision. Strengthening clinical correlation studies will also be pivotal in realizing their potential for drug development and personalized medicine.

## 5 Frontier Technologies for the Construction of Neural Organoids

While traditional organoids generation methods are well‐established and highly reproducible, they often fall short in capturing the full complexity of neural tissue development due to limitations in size, cell composition, and developmental stages. The construction of neural organoids has been significantly advanced by the application of frontier technologies, which aim to better mimic the complexity and functionality of native neural tissues (**Table** **5**). Microfluidics and organoid technologies have been developed to precisely regulate the microenvironment during organoid construction. Meanwhile, Micro‐patterning and Bioprinting offer novel approaches for optimizing the structural design of organoids. Although these technologies currently have certain limitations, they provide significant impetus for enhancing the biomimetic fidelity of organoid structure and function.

**Table 5:** Advantages and limitations of different technologies for organoid generation.

Generation method	Description	Advantages	Limitations
Generation by signaling molecules	Utilizes the self‐organizing properties of stem cells, with the addition of specific concentrations of inducers/nutrients at different stages to generate organoids that partially mimic the structural and physiological characteristics of native tissues.	Well‐established culture protocols with high reproducibility. Capable of simulating a wide range of tissue types through adjustable induction protocols. Suitable for high‐throughput screening applications.	Limited organoid size. Homogeneous cell composition. Lagging developmental stages.
Microfluidics and Organoids‐on‐Chip	Incorporates micron‐sized structures for 3D culture and integrates microsensors.	Precise control over the cellular microenvironment, including fluid flow, oxygen gradients, and nutrient delivery, thereby more accurately mimicking in vivo physiological conditions.	Complex instrumentation. Limited throughput, restricting applications such as large‐scale drug screening.
Micro‐patterning	Spatially arranges cells at the micrometer scale using micropatterned ECM or physical structures to guide self‐organization of organoids.	Simple and precise control of initial cell arrangement and distribution, guiding cells to grow and differentiate in specific patterns to better simulate tissue architecture.	Lacks dynamic environment and complex interactions among multiple cell types. Tends to form 2.5D structures rather than 3D ones. Limited maturity and functional complexity of the organoids.
Bioprinting	Combines biofunctional materials with growth factors and multiple cell components to fabricate 3D organoids with desired tissue structures and spatial distributions using a bioprinter.	Facilitates the creation of complex and larger scaled biomimetic 3D tissue structures.	Further improvements needed in resolution and precision. Potential reduction in cell viability during the printing process.

### 5.1 Microfluidics and Organoids‐on‐Chip

**Figure 7:**
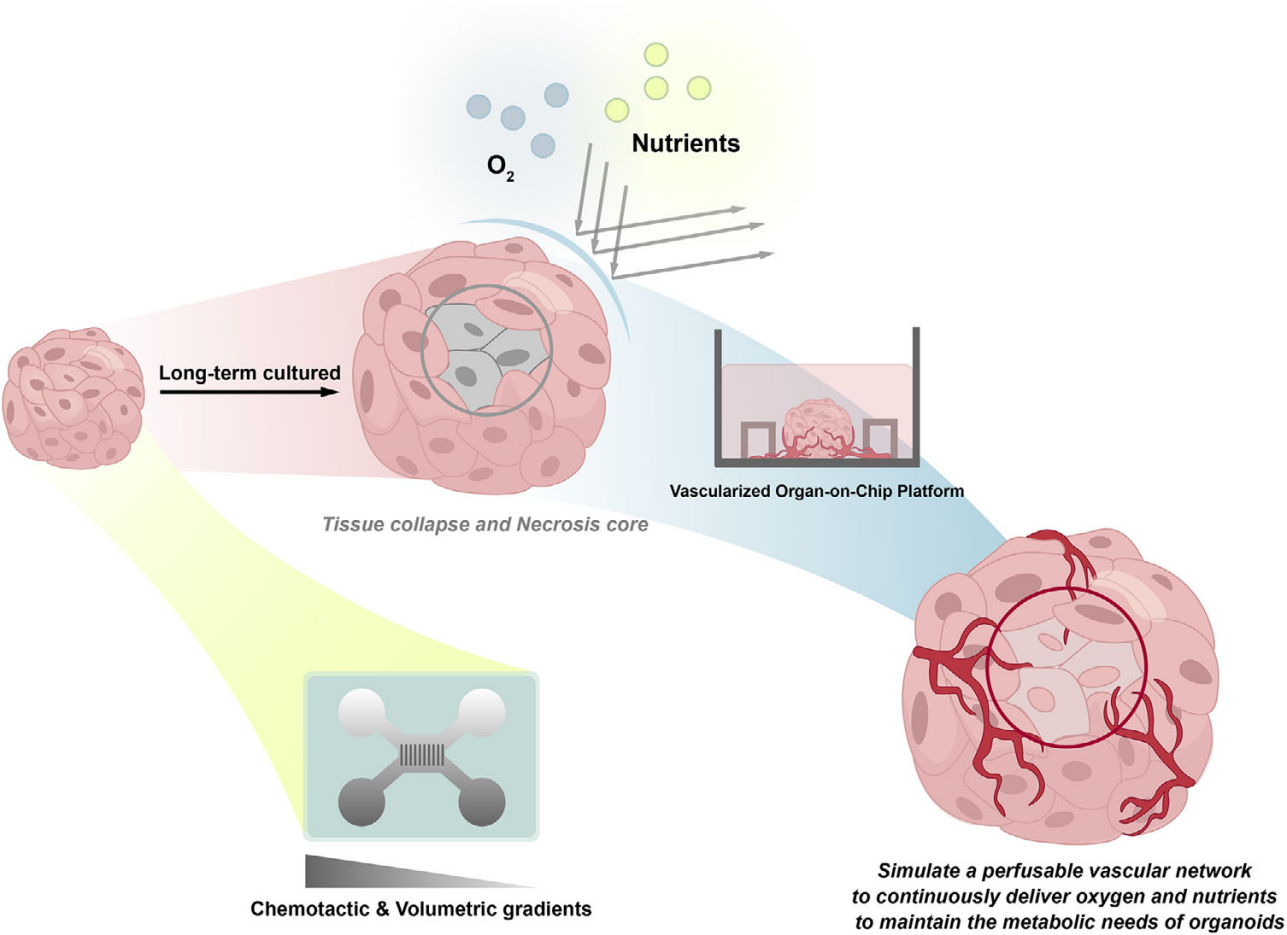
Organ‐on‐Chip technology provides new opportunities for the optimization of organoid culture protocols. Created by Adobe Illustrator 2024.

In recent years, research on neural organoids has made significant progress. By utilizing a variety of biomaterials and bioreactors, along with advanced gene editing technologies, researchers have gradually developed neural organoids that exhibit higher maturity and refinement, more closely resembling their natural state. However, traditional 3D organoid culture systems, which rely on ECM gels, are limited by the conditions of the culture environment. These organoid construction systems still face challenges, including homogeneous cell composition, the inability to simulate vascular perfusion, and the central tissue necrosis during long‐term culture.^[^ ^93^, ^238^ ^]^ Microfluidics is a scientific technology that precisely controls and manipulates micro‐scale fluids within micro‐ and nano‐scale environments. Organ‐on‐a‐chip is a technology developed from microfluidic and 3D printing technologies to effectively simulate the human physiological environment and create micro in vitro models of organ physiological functions.^[^ ^239^, ^240^ ^]^ Organ‐on‐a‐chip technology overcomes the limitations associated with traditional organoid construction methods (**Figure** **7**). Firstly, the microchannels within the chips facilitate the establishment of morphogen gradient microenvironments essential for in vitro embryonic organogenesis, thereby transcending the paradigm barrier that separates organoid construction from actual tissue development.^[^ ^241^ ^]^ The researchers developed a organoids‐on‐chips that enable the simultaneous maintenance of relative and/or orthogonal developmental morphogen gradients to induce hESCs by precisely controlling fluid flow. This setup simulates the spatiotemporal chemical environment encountered during embryonic neural tube development, allowing hESCs to differentiate into motor neurons and form a distribution of neural subtypes that closely resembles the neural tube.^[^ ^242^ ^]^ Researchers have developed a microfluidic platform that effectively reduces heterogeneity in brain organoid size through a streamlined assembly and culture process. This platform minimizes losses during transfer and mitigates the formation of hypoxic cores by utilizing an air–liquid interface culture. It has been employed to investigate the effects of prenatal cannabis exposure on early human brain development.^[^ ^243^ ^]^ Furthermore, other studies have demonstrated that by inserting hESCs clusters into compartments of a microfluidic chip and filling these compartments with a collagen‐laminin‐based hydrogel (Matrigel), the model can simulate cortical folding during brain development and facilitate the analysis of the physical and biological mechanisms associated with this process.^[^ ^244^ ^]^

Numerous successful cases of constructing region‐specific brain organoids have been reported; however, most brain organoids cannot be cultured for extended periods due to the absence of a vascular network that continuously supplies nutrients essential for tissue survival and maturation. While there are studies focused on developing brain‐vascular organoid fusion systems by simultaneously integrating vasculature and microglia‐like cells within brain organoids, functional vascular perfusion has yet to be achieved.^[^ ^82^ ^]^ To this end, some researchers have developed a vascularized organ‐on‐chip platform that sustains the metabolic needs of brain organoids by simulating a perfusable vascular network. This innovation facilitates the delivery of drugs and immune cells, addressing the challenges posed by long‐term culture of brain organoids, which often lead to tissue collapse and necrosis core due to insufficient nutrients and oxygen. Furthermore, this approach enhances the reproducibility and batch‐to‐batch consistency of brain organoid construction. Meanwhile, the porous membrane‐based chip serves as an interface between compartments, allowing for both indirect and direct interactions, and is utilized to simulate structures such as the blood‐brain barrier.^[^ ^243^, ^245^, ^246^, ^247^ ^]^

### 5.2 Generation of Neural Organoids Based on Bio‐Engineering

#### 5.2.1 Application of Bio‐Functional Materials in Organoids

**Figure 8:**
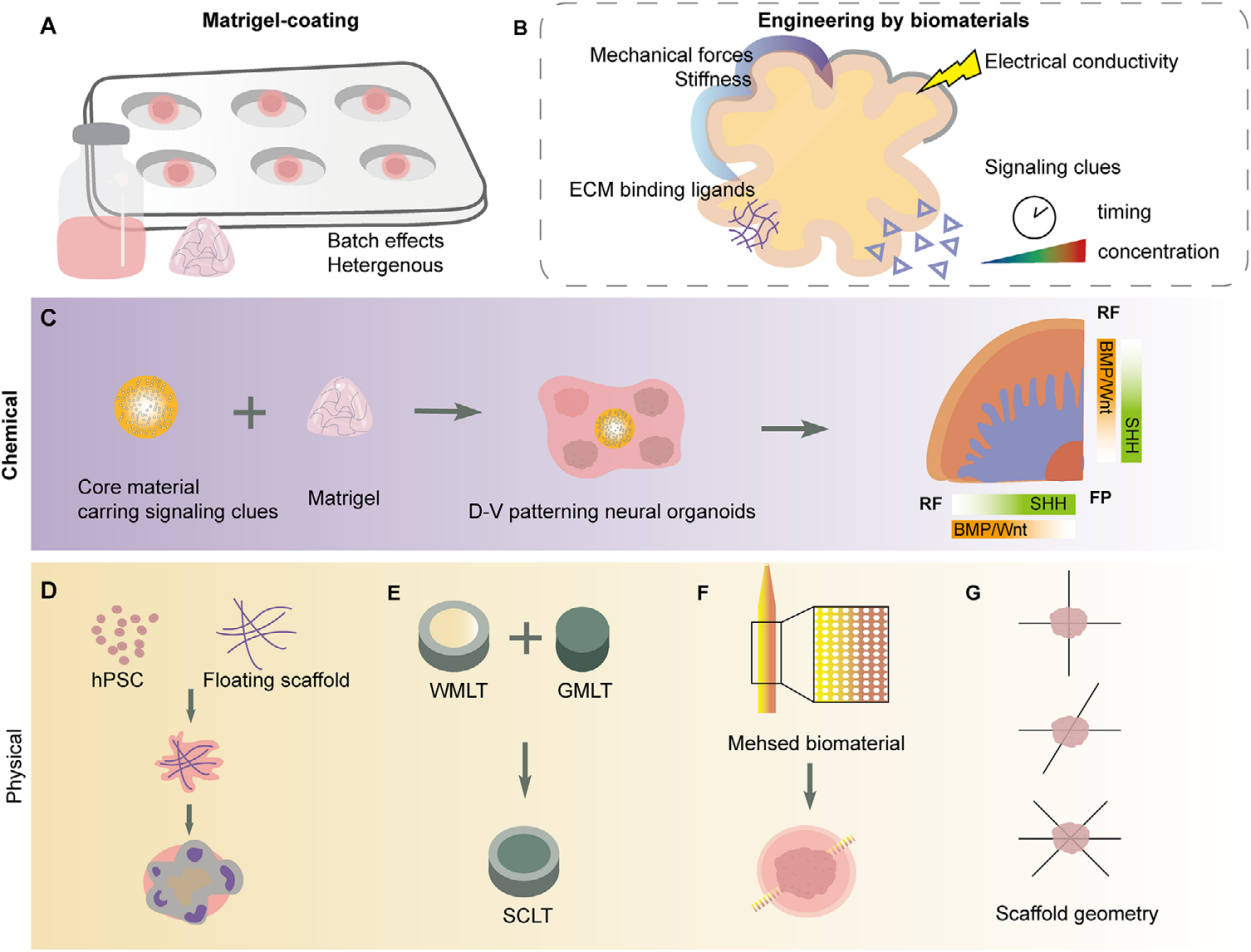
Effects of biomaterials on neural organoids. A) Traditional neural organoids generation by Matrigel. B) Chemical or physical effects of biomaterials on engineered biomaterials. C) Controlled release of signaling clues by biomaterial shapes the developmental patterning of neural organoids. D) PLGA scaffold promotes neural maturation in organoids. E) Biomaterials for architecting neural‐like organs. F) Meshed biomaterial for preventing necrotic cores. G) Geometry of scaffold regulates stem cell self‐organization. Created by Adobe Illustrator 2024.

Both intracellular and extracellular signals collaboratively guide the formation of organoids and tissue structures. The interface cues within the culture environment can exert dimension‐dependent effects on the morphology of neuroepithelial organoids, including the development of cavity structures and the formation of the floor plate.^[^ ^248^ ^]^ In protocols of organoid generation, researchers typically provide an ECM environment through the coating of Matrigel to facilitate the expansion and differentiation of neuroectodermal cells. However, Matrigel is a complex mixture of ECM proteins, growth factors, and other components derived from Engelbreth–Holm–Swarm (EHS) mouse sarcoma. Different batches of Matrigel can have varying concentrations of key components like laminin, collagen IV, and growth factors. These differences can lead to inconsistent cell adhesion, proliferation, and differentiation, ultimately affecting organoid formation. Additionally, the way Matrigel is thawed, mixed, and applied to culture dishes can affect its properties and the subsequent cell behavior.^[^ ^249^ ^]^ This introduces animal‐derived components into the resulting neural organoids, leading to batch effects among samples (**Figure** **8**A). To address these issues, various biomaterials have been developed to provide stable and effective ECM signals. These biomaterials can be natural (such as collagen, hyaluronic acid) or synthetic (such as polycaprolactone and polyethylene glycol), as well as metals and ceramics, all aimed at regulating cell fate and behavior within organoids. Diversity biomaterials has been well characterized, with many now commercially available. Among these, non‐animal‐derived gel materials, such as alginate, have been shown to generate standardized neural fates.^[^ ^250^ ^]^ Effective and extensively researched biomaterials include synthetic hydrogels derived from decellularized ECMs (dECMs) sourced from neural tissues such as the brain, spinal cord, and retina, as well as from placental.^[^ ^251^, ^252^, ^253^ ^]^ The dECMs are obtained through decellularization of tissues, followed by adjustments in temperature and pH to dissolve the matrix, then apply reassembly of molecular intra‐linkages to form synthetic hydrogels. These hydrogels preserve the composition and structure of the natural extracellular matrix, are rich in bioactive components such as collagen IV and laminin, and exhibit excellent biocompatibility and bioactivity. These properties promote cell adhesion, proliferation, and differentiation, thereby providing a favorable environment for the growth and maturation of organoids. Researches indicate that these hydrogels derived from tissues can enhance neuronal populations and promote neurogenesis.^[^ ^88^ ^]^ Additionally, it has been found that dECMs from neonatal animals significantly improve neurite outgrowth and elongation in neural organoids compared to those derived from adult animals.^[^ ^252^ ^]^ Organoids encapsulated in these dECMs also demonstrate remarkable capabilities for tissue integration and functional reconstruction post‐transplantation.^[^ ^252^, ^254^ ^]^

The construction of patterned organoids can be achieved through the controlled release of morphogens or induction signals from biomaterials. Numerous studies have demonstrated that tissue‐specific modifications to biomaterials, such as the incorporation of signaling molecules, can induce neurogenesis in engineered tissues. Various growth factors, including neurotrophins such as NT3, Brain‐derived neurotrophic factor (BDNF), Glial cell line‐derived Neurotrophic Factor (GDNF), and Ciliary neurotrophic factor (CNTF), as well as BMPs, have been utilized to promote the differentiation of neurons or glia. Additionally, FGF and epidermal growth factor (EGF) facilitate the proliferation of progenitor cells.^[^ ^229^ ^]^ Research indicates that the concentration, location, and timing of signaling pathway ligands dictate cell fate, and biomaterials can be finely tuned to modulate these parameters. A neural tube model was constructed using laminin and PEG hydrogel scaffolds for the dose‐controlled release of retinoic acid, successfully inducing neuroepithelial tissues with a distinct D–V structure.^[^ ^255^ ^]^ Researchers also employed porous chitosan microspheres as a slow‐release Shh agonist to spatially regulate signaling molecules, in combination with Matrigel, to create distinct D–V progenitor cell domains ^[^ ^256^ ^]^ (Figure 8C).

The morphology and development of organoids can be jointly regulated by physical factors (Figure 8B). Mechanical forces, electrical stimuli, and magnetic cues have been shown to influence neurogenesis and neural development in neural organoids. Biomaterials can also be employed to investigate how physical properties, such as extracellular mechanical forces, impact the morphology and patterning of organoids. By introducing micron‐scale poly(lactide‐co‐glycolide) (PLGA) scaffold materials into organoids, one can enhance nutrient and oxygen channels while promoting the elongation of EBs, thereby augmenting the formation of the neuroectoderm through physical polarity modulation (Figure 8D).^[^ ^257^ ^]^ Meshed biomaterials also exhibit abilities as flow channel structures that inhibit the formation of necrotic cores within neural organoids ^[^ ^150^ ^]^ (Figure 8F). Fattah et al. developed neural tube organoids using PEG and laminin as ECM, combined with signaling molecules. They create devices that enable the actuation of organoids through ECM, their findings revealed that mechanical forces can enhance organoid patterning.^[^ ^258^ ^]^ Mechanical forces also play a feedback regulatory role in the morphogenesis of retinal organoids.^[^ ^259^ ^]^ Moreover, melt electrospinning writing has been utilized to produce tunable grid scaffolds, where the scaffold geometry has been shown to be a critical determinant of stem cell self‐organization (Figure 8G). Controlled curvature of tissue growth guides the positioning and size of lumens in neural organoids.^[^ ^260^ ^]^

#### 5.2.2 Bio‐Manufacturing Techniques

The integration of biomaterials with 3D printing technology enables the construction of complex spatial organizations, facilitating the regional design and assembly of neural organoids, as well as the generation of large‐scale organoids. The inks used in 3D printing are often bifunctional materials that can be infused with growth factors and various cells. When selecting biomaterials, key considerations include not only biocompatibility but also cell adhesion properties and their ability to promote cell proliferation and differentiation. Among the many functional materials, gelatin‐ and fibrin‐modified gels have emerged as widely utilized bioinks in 3D printing applications.^[^ ^261^ ^]^ Some of these materials, due to their high stability and mature production lines, have been commercialized. Numerous studies have enhanced the cell compatibility of bioprinted constructs by incorporating cell‐adhesive peptides, such as arginine–glycine–aspartic acid (RGD), into these bioinks.^[^ ^262^, ^263^ ^]^ In addition to approaches utilizing bioactive additives, some studies have enhanced cell adhesion by engineering the physical architecture of scaffolds. Li et al. employed tetrapod‐shaped zinc oxide (t‐ZnO) microparticles as templates to create microstructured alginate scaffolds via 3D printing. This method introduces interconnected channels and textured surfaces into the alginate, significantly improving neural cell adhesion and growth through physical design.^[^ ^264^ ^]^ Thermosensitive ECM hydrogels represent an excellent choice for 3D printing bioinks. In addition to the chemical properties of bioinks, physical characteristics, such as elastic modulus and shear forces acting on encapsulated cells, significantly influence cellular fate post‐printing. Neurons exhibit heightened sensitivity to the mechanical properties of the ECM. Studies indicate that the cytoskeleton of neurons in the DRG responds to substrate stiffness gradients.^[^ ^265^ ^]^ Ideally, bioinks for organoid engineering should closely mimic human tissue properties to promote adaptive cell growth. For instance, the elastic modulus of spinal cord tissue ranges from 200 to 600 kPa, and hydrogels with an adjusted modulus of ≈400 kPa have been shown to effectively induce axonal regeneration in spinal cord models.^[^ ^266^ ^]^ Multiple studies have confirmed that hydrogels with an elastic modulus of 1–10 kPa are most conducive to stem cell differentiation into neural tissue.^[^ ^267^, ^268^, ^269^ ^]^ When constructing neural organoids or transplanting engineered tissues to promote neural regeneration, it is essential to adjust conditions according to the research objectives, aligning them with the desired cellular fate. Furthermore, the shear stress experienced during 3D printing affects the viability of the encapsulated cells, with higher shear forces correlating negatively with cell viability.^[^ ^270^ ^]^ The viscosity of the bioink, hardware conditions such as extrusion pressure and nozzle diameter, can impact shear stress collectively.^[^ ^271^ ^]^ Accordingly, optimizing the physical properties of biomaterials alongside the hardware and software settings of 3D bioprinters is crucial for effectively simulating natural tissues in the engineering of neural constructs.

Researchers have developed a variety of bioprinting methods for organoid generation and drug screening (**Table** **6**). Among these, microfluidic extrusion bioprinting allows for the precise printing of NSC aggregates, which can further differentiate into highly viable mature neurons.^[^ ^272^ ^]^ Handheld extrusion bioprinting technology has been employed to recreate the structural organization of cortical layers, with cortical neurons encapsulated in distinct layers that form neural connections post‐differentiation in vitro.^[^ ^262^ ^]^ Digital light processing bioprinting offers high printing resolution suitable for investigating cellular interactions and has been utilized in high‐throughput drug screening platforms for glioblastoma models.^[^ ^131^ ^]^ Multi‐material extrusion bioprinting has been widely applied to the complex architecture of SCLT, enabling the precise positioning of different cell types and growth factors to create biomimetic structures (Figure 8E).^[^ ^273^ ^]^ Gai et al. developed a biomimetic material system composed of gelatin, alginate, and fibrinogen for 3D‐printed NPC constructs.^[^ ^274^ ^]^ This system demonstrated integration capabilities with host tissues and provided a controllable and reproducible platform for neural stem cell therapy, and holds significant promise for the modular assembly of brain organoids. Additionally, 3D printing can be integrated with other advanced techniques to achieve more accurate structures or enhanced functionalities. Cadena et al. utilized 3D bioprinting to fabricate a highly tunable microchanneled scaffold based on gelatin methacrylate (GelMA). The scaffold design included interconnected endothelialized channels and a top‐loading channel, enabling precise control over organoid development and patterning. This platform demonstrated exceptional reproducibility and supported the long‐term co‐culture of organoids with vascular cells, creating highly complex and robust in vitro platforms.^[^ ^275^ ^]^ Recent studies have utilized 3D printing to create high‐throughput, adjustable, and reproducible scaffold materials that support the mutual infiltration of organoids and vasculature.^[^ ^89^ ^]^ By combining biofunctional materials with microfluidic systems, researchers have achieved more mature and reproducible brain organoids.^[^ ^88^ ^]^

**Table 6:** Bioprinting methods for organoid generation.

Bioprinting modality	Working principle	Characteristics	Application	Refs.
Photocuring Bioprinting	Uses light to cure photosensitive bioinks.	High precision, rapid prototyping, suitable for complex structures.	Fabricating tissue‐engineered scaffolds or organoids with fine structures.	[276]
Extrusion‐based Bioprinting	Extrudes bioink through mechanical pressure layer by layer.	Suitable for high‐viscosity bioinks, simple operation, and cost‐effective.	Fabricating tissue‐engineered scaffolds for cartilage, skin, and bone.	[277]
Inkjet‐based Bioprinting	Uses thermal or acoustic waves to eject bioink droplets onto a substrate.	Non‐contact printing, suitable for low‐viscosity bioinks, high precision.	Fabricating models for skin, vascular, and neural tissues.	[278, 279]
Laser‐assisted Bioprinting	Uses lasers to transfer bioink from a donor substrate to a receiver substrate.	High precision, noncontact printing, suitable for precise cell positioning.	Fabricating complex cellular patterns and tissue‐engineered structures.	[280]
Microfluidic‐based Bioprinting	Uses microfluidic technology to control the flow and deposition of bioinks.	High precision, capable of multi‐material printing, suitable for complex structures.	Fabricating organoids and microphysiological systems.	[281]
Acoustic‐based Bioprinting	Uses acoustic waves to precisely eject bioink droplets onto target positions.	Noncontact printing, suitable for high‐precision and complex structures.	Acoustic‐based bioprinting has been applied to the construction of meniscus‐like tissues and tumor spheroid‐stroma composites.	[282]
Embedded 3D Bioprinting	Prints structures within a supportive gel to provide mechanical support.	Suitable for complex geometries and structures.	Embedding cells in biomaterials which is ideal for shaping lumen‐like structures.	[275]
Multi‐material Bioprinting	Combines multiple bioinks or materials to print complex structures with multiple functions.	Mimics the heterogeneity and complexity of natural tissues.	Fabricating multifunctional tissues and organ models.	[283]

In addition to 3D printing technology, researchers have developed various bio‐material integrated shaping technologies for organoid structures. Solvent casting involves dissolving biomaterials in a specific solvent, mixing a cell suspension containing organoids with the solution, and casting it into a mold to form a 3D culture system after the solvent solidifies.^[^ ^284^ ^]^ This technique is easy to perform and can produce scaffolds with high porosity and interconnected pores. It can also be used to prepare scaffolds in various shapes and sizes to meet the needs of different organoid cultures. Electrospinning technology can precisely control fiber diameter, alignment, and density to mimic the fibrous structure of the extracellular matrix. It can be used to prepare various tissue engineering scaffolds, such as neural, bone, and muscle fiber scaffolds, and has been applied in the construction of artificial tissues like heart valves.^[^ ^285^ ^]^

## 6 Limitations of Recent Neural Organoids

Despite significant breakthroughs in the generation of neural organoids and the development of disease models based on these systems over the past decade, challenges remain in this field. The maturity of existing neural organoids, in terms of both cellular composition and network‐level events, aligns only with the developmental stage of the fetus.^[^ ^118^, ^286^ ^]^ This limitation prevents researchers from utilizing these organoids as broadly representative models for the comprehensive investigation of the pathophysiology and functionality of neurodegenerative diseases prevalent in the aging human population. Furthermore, neural organoids fail to accurately reflect the complete tissue morphology of human brain structures, which possess a complex and intricate multilayer architecture, including six layers of the cortex. Existing brain organoids only achieve simplistic layering, forming deeper and upper layers outside the OSVZ.^[^ ^18^, ^287^ ^]^ Similarly, SCOs do not replicate the synchronous development of the R–C, D–V, and M–L body axes, and the diversity of neuronal and non‐neuronal cells is far less than that of the human CNS. In organoid construction, researchers predominantly focus on the role of signaling molecules in determining cell fate, while factors such as physical polarity and spatial positioning significantly influence organoid morphogenesis and tissue architecture. Recent studies have begun to acknowledge the effects of physical modulation, such as symmetry breaking, on organoid developmental patterns.^[^ ^288^ ^]^ Constructing neural organoids that accurately mimic the in vivo cellular microenvironment is highly complex, particularly in terms of building tissue–tissue interfaces, which are crucial for studying key developmental stages and pathological mechanisms. Organoids lack a functional immune environment, which limits their potential in transplantation applications.^[^ ^289^ ^]^ Although attempts have been made to address this issue using co‐culture or gene‐editing techniques, the immune microenvironment of organoids remains difficult to fully establish. Additionally, issues such as the disorganized tissue structures caused by multiple neurodevelopmental centers and limited morphological sizes remain urgent challenges to be addressed.

## 7 Conclusion and Outlooks

**Figure 9:**
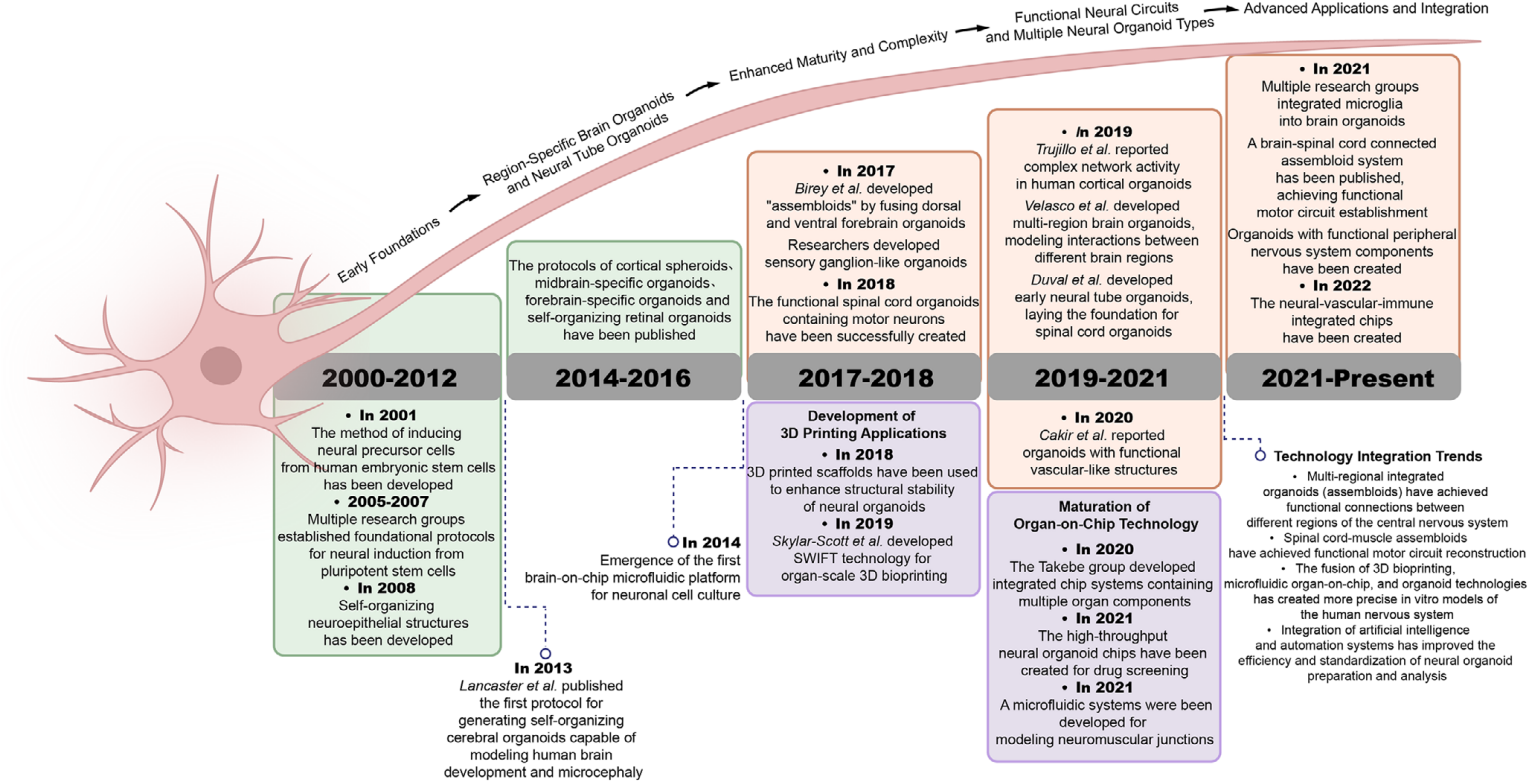
The timeline of core neurological organoid technology development. Created by Adobe Illustrator 2024.

Neural organoids provide a powerful platform for studying human neural development and neurological diseases. Various organoids representing specific regions of the CNS have been generated. Researchers have also highlighted the promising applications of efficient subtype specification in disease modeling. For instance, by activating the WNT and SHH signaling pathways, iPSCs can be induced to differentiate into 5‐HT‐organoid, which hold great promise for investigating neuropsychiatric symptoms in AD patients.^[^ ^78^ ^]^ These in vitro culture systems provide an efficient and highly relevant platform for investigating human neural development, tissue structural formation, and neurological disorders. Some limitations of neuroectodermal organoids, such as the lack of vascular system and microglia have been addressed. Emerging technologies, including organoid‐on‐a‐chip and tissue engineering, offer novel approaches for the generation of neural organoids (**Figure** **9**). Advanced research methodologies, such as spatial transcriptomic,^[^ ^290^ ^]^ MEA technology,^[^ ^233^ ^]^ and tissue clearing combined with 3D imaging,^[^ ^291^ ^]^ offer novel insights into the cellular interaction and communication mechanisms of organoids, as well as enable comprehensive analysis of their tissue organization and structural complexity.

The application of biomaterials in the construction of neural organoids is evolving rapidly, giving rise to new paradigms in organoid generation. Smart materials that respond to external stimuli, including temperature, pH, or light, are being employed to control the release of growth factors or gene carriers, enabling temporal and spatial regulation of cellular behaviors.^[^ ^292^ ^]^ In conventional organoid construction protocols, agonists or antagonists of morphogenetic signaling pathways are added to the culture medium at different stages to sequentially induce cell fate decisions. Some layered materials, such as graphene and layered double hydroxides (LDH), have become promising tools for controlling stem cell fate. Graphene offers high biocompatibility, stability, scalability, and functionalization capabilities that promote specific cellular responses (e.g., cell adhesion and growth).^[^ ^293^ ^]^ Additionally, its conductivity enables combined bioelectrical stimulation to induce a more mature phenotype in NSCs.^[^ ^294^ ^]^ These properties suggest that graphene/carbon nanotubes, when combined with hydrogels, hold potential for applications in neural organoids. Recent findings indicate that LDH exerts bidirectional regulation on mouse ESCs in a time‐dependent manner by activating Lif receptor or Ptch1 on the cell membrane.^[^ ^295^ ^]^ It is conceivable that advancements could enable the design of biomaterials that precisely and conditionally bind to receptors, facilitating a streamlined approach to organoid generation. During neurodevelopment, the subtype specification, migration, and axonal projections of NPCs are regulated not only by the spatiotemporal control of morphogen gradients but also by cellular architecture, cell–cell interactions, and paracrine signaling.^[^ ^296^ ^]^ Conventional organoid culture systems often provide an isotropic environment, leading to uniform or stochastic self‐assembly of cell types. Biomaterials, however, can mediate diverse signaling cues in neurodevelopment, such as the mechanical properties of the extracellular matrix, microarchitecture, and cellular alignment,^[^ ^297^ ^]^ thereby offering multidimensional regulation of the developmental complexity of neural organoids. Additionally, magneto‐responsive hydrogels have introduced innovative applications in organoid fabrication. A research team has developed the spatially patterned organoid transfer (SPOT) technology, enabling controlled lifting, transport, and assembly of organoids.^[^ ^298^ ^]^ This technique holds significant potential for fine‐tuning the architectural arrangement of organoid tissues. Beyond optimizing neural organoid construction, functional materials have promising applications in the functional study of organoids. For instance, a mesh electrode array based on conductive hydrogels can non‐invasively interface with floating neural organoids in culture, allowing for real‐time recording of neural signaling and illuminating the dynamics of neural networks.^[^ ^299^ ^]^

The integration of AI technologies with organoid techniques has the potential to deeply elucidate cellular information and optimize construction protocols, thereby offering high‐throughput predictive and diagnostic tools for personalized medicine. Recent studies combining single‐cell RNA sequencing with machine learning approaches have provided an in‐depth analysis of cellular heterogeneity within organoids, offering valuable insights for the precise construction and functional investigation of organoids.^[^ ^300^ ^]^ AI's capabilities in big data processing have improved researchers' ability to derive biological insights from organoid models.^[^ ^301^ ^]^ Ajinkya et al. constructed pancreatic cancer organoids, combined with live‐cell imaging technology to monitor the interactions between organoids and immune cells in real‐time, and analyzed the morphological changes of organoids using deep learning algorithms, providing a standardized benchmark dataset for cancer immunotherapy research.^[^ ^302^ ^]^ Luca et al. developed an AI‐based segmentation and analysis pipeline for high‐field magnetic resonance monitoring of cerebral organoids, enabling non‐invasive real‐time monitoring, which has significant applications in drug screening and developmental studies.^[^ ^303^ ^]^ Patient‐derived organoids require qualitative analysis through methods like immunofluorescence and single‐cell sequencing, however, recent studies have utilized bright‐field imaging and deep learning to reveal morphological heterogeneity in colorectal cancer, offering novel insights into disease mechanisms.^[^ ^304^ ^]^ These studies not only provide technical support for non‐invasive monitoring of organoids but also advance drug screening and developmental research by offering innovative approaches. In addition to analyzing data related to cell identity and structural characteristics in organoids, researchers are now employing AI to optimize organoid culture conditions. This approach facilitates the screening and design of both extracellular and intracellular signals, thereby improving the efficiency and consistency of organoids. The ECM significantly influences cell behavior, and optimizing suitable gels for cell proliferation and differentiation is a time‐consuming process that often requires multiple iterations to adjust individual experimental parameters. Integrating machine learning to examine how different polymers affect stem cell behavior and organoid generation can facilitate predictions regarding the structure and properties of hydrogels, thereby expediting the development of custom matrix gels.^[^ ^305^ ^]^ This approach not only enhances the efficiency of organoid research but also opens new avenues for personalized medicine. The organoid construction process typically involves the induction of various morphogens and growth factors, necessitating lengthy experimental cycles to adjust the composition, induction duration, and concentration of signaling molecules. A study has reported that deep learning neural networks, combined with high‐throughput imaging flowcytometry techniques, can accurately predict the differentiation direction of NSCs at early stages. This approach is applicable for screening regulatory factors, including functional biomaterials.^[^ ^306^ ^]^ This technology may also be relevant for predicting the fate of terminally differentiated neuronal subtypes and offers significant insights for the generation of region‐specific or highly mature organoid. The development of neural organoids may have the potential to advance AI systems, particularly in the field of brain‐inspired computing. Cai et al. use organoids as physical substrates for reservoir computing, leveraging their inherent neural network architecture and dynamic properties to facilitate information storage, processing, and output.^[^ ^307^ ^]^ By mimicking the information processing mechanisms of the human brain, neural organoids may significantly enhance the performance of AI systems. The integration of AI and organoid technologies is continually expanding the frontiers of neuroscience research. From extracting cellular information and optimizing functions to applying brain‐inspired computing, these emerging directions offer unprecedented opportunities for personalized medicine and neuroscience. Future research could further combine AI to develop more efficient and smarter platforms for organoid cultivation and analysis, providing novel solutions for modeling and treating complex diseases.

With the advancement of organoid engineering and its integration with AI, the increasing complexity of organoids in recent years may bring them closer to replicating the information‐processing capabilities of the human brain. In this context, assessing their conscious state and avoiding unintentionally causing suffering (e.g., during drug screening processes) becomes an important consideration. Additionally, transplanting neural organoids into animals may alter the animals' conscious state, raising ethical concerns.^[^ ^308^ ^]^ To address ethical issues in the cultivation of more complex and complete neural organoids in the future, it is essential to establish interdisciplinary collaboration among philosophy, neuroscience, and ethics early on, ensuring the synchronization of technological advancement with ethical frameworks.

The next version of organoid technology may involve the integration of above emerging techniques to provide high‐precision screening platforms for genetic diseases. Chimeric organoids will serve as scalable systems for high‐throughput investigations into individual genetic variations during brain development and disease processes, thereby advancing precision medicine.^[^ ^17^ ^]^ It is also worth anticipating that these technologies will facilitate the in‐plate construction of CNS tissue, offering guidance for non‐rejecting transplantation strategies for neural repair following injury.

## Conflict of Interest

The authors declare no conflict of interest.

## Author Contributions

R.Z. proposed the topic of the review and revised the manuscript. R.H., F.G., and L.Y. investigated the literature and prepared the manuscript. H.C. participated in the figure and Endnote library preparation. R.H., F.G., and L.Y. contribute equally to this work.
